# AI-based integration of ECG biomarkers for assessing cardiac risk in type 2 diabetes mellitus with comorbid conditions for patient stratification

**DOI:** 10.3389/fmed.2025.1646495

**Published:** 2025-09-08

**Authors:** Symeon Savvopoulos, Haralampos Hatzikirou, Herbert F. Jelinek

**Affiliations:** ^1^Department of Mathematics, Khalifa University of Science and Technology, Abu Dhabi, United Arab Emirates; ^2^Center for Interdisciplinary Digital Sciences (CIDS), Department Information Services and High Performance Computing (ZIH), TUD Dresden University of Technology, Dresden, Germany; ^3^Department of Medical Sciences and Health Engineering Innovation Center, Khalifa University of Science and Technology, Abu Dhabi, United Arab Emirates; ^4^Health Engineering Innovation Group, Khalifa University of Science and Technology, Abu Dhabi, United Arab Emirates

**Keywords:** electrocardiography (ECG), diabetes, arrhythmia medications, cardiovascular risk, hypertension disease progression, screening

## Abstract

**Introduction:**

The increasing prevalence of type 2 diabetes mellitus (T2DM) requires improved early detection strategies that integrate demographic, clinical, physiological, and pharmacological data. Electrocardiographic (ECG) biomarkers offer a non-invasive means to assess diabetes-related cardiac risk, particularly in individuals with hypertension (HT) and cardiovascular disease (CVD) comorbidities of diabetes.

**Methods:**

ECG data from 581 subjects were categorized by glycemic status (healthy, prediabetes, T2DM) and comorbidities. Demographic, clinical, and pharmaceutical data were merged with 10 s and 5 min ECG recordings. SMOTE was used to correct class imbalance. Support Vector Machines (SVM) performed best among machine learning classifiers. Classification accuracy, sensitivity, specificity, and AUC were computed using 5-fold cross-validation. Feature importance was assessed through permutation analysis to identify the most discriminative ECG and medication-related predictors.

**Results:**

T2DM patients, particularly those with HT and CVD, exhibited significant prolongation of QTc (10 s), QTd (10 s and 5 min), and PQ intervals, as well as changes in the QRS-Axis, indicating increased arrhythmic risk and electrical remodeling (*p* < 0.001). Antihypertensive and lipid-lowering medications influenced QRS-Axis and PQ intervals, while antidepressant use was associated with QTd dispersion (*p* = 0.010). Classification accuracy ranged from 0.64 to 0.88. Five-minute ECGs provided higher accuracy (~0.88) when medication data were included, while 10-s ECGs performed well in treated patients (~0.86–0.88).

**Discussion:**

This study shows that ECG-based, AI-driven screening captures the interaction between comorbidities, medication use, and cardiac electrophysiology. Integrating ECG biomarkers with medication data improved T2DM risk classification, enabling better treatment outcomes based on clinical use of non-invasive methods for risk classification.

## 1 Introduction

Type 2 diabetes mellitus (T2DM) is a complex and progressive disease characterized by chronic hyperglycemia resulting from insulin resistance, impaired insulin secretion, and increased gluconeogenesis (1). The global burden of T2DM continues to increase despite improved medication and lifestyle intervention opportunities, with 828 million individuals presenting with diabetes worldwide, rising from 7% in 1990 to 14% in 2022. However, the number may be much higher as approximately 59% of people with diabetes over 30 years of age are either not identified in the healthcare systems or untreated, highlighting healthcare access and treatment inadequacies, possibly due to a lack of screening opportunities and early diagnosis based on biomarkers other than fasting glucose or HbA1c. Negative lifestyle changes, urbanization, and aging populations are driving the increase in T2DM, emphasizing the need for public health measures to promote prevention, early diagnosis, and treatment (2–6). Screening approaches also need to consider that T2DM often coexists with hypertension (HT) and cardiovascular disease (CVD), leading to a more complex clinical presentation that compounds morbidity and healthcare costs. Recent advances in understanding the pathophysiology of T2DM have highlighted the significant heterogeneity in metabolic phenotypes, ranging from isolated insulin resistance to severe *β*-cell dysfunction, which influences the progression of T2DM and the occurrence of comorbidities on an individual level and hence requires individualized reviews and treatment. Numerous studies have shown the connection between T2DM and genetics, environment, and lifestyle factors such as nutrition, exercise, and inactivity (7). Liver, pancreas, and muscle pathology in addition to increased levels of cholesterolcan contribute to the development of T2DM. Characterizing the contribution of individual factors that can affect T2DM and its progression, as well as the occurrence of comorbidities, could make treatment more effective (8). However, significant gaps remain in integrating these approaches for patients with comorbidities, particularly in resource-limited settings. The varying disease course and therapy responses of T2DM are associated with changed metabolic interactions, depending on the presence or absence of comorbidities (9). These variations in metabolic processes and their etiology may help identify patients at risk for T2DM in primary and secondary prevention studies, as well as those who can benefit from personalized interventions.

T2DM has many pathophenotypes and management approaches. Effective treatment often requires the use of angiotensin-converting enzyme (ACE) inhibitors, angiotensin II receptor blockers (ARBs), and thiazide diuretics to manage concomitant diseases such as hypertension and cardiovascular risk in T2DM patients (10). Statins and aspirin are prescribed to T2DM patients to treat dyslipidemia (11). The increased prevalence of hypertension in T2DM patients highlights the need for better monitoring that addresses oxidative stress and inflammation, two major contributors of poor glycemic control (11, 12). Although we have a better understanding of the T2DM and disease progression including comorbidities there are few studies on the classification of T2DM comorbidities. Recent research has shown biomarker alterations with the transitions from normal blood sugar levels to prediabetes and T2DM, especially associated with comorbidities. Key alterations encompass mitochondrial-derived peptide MOTSc, as well as reduced glutathione, glutathione disulfide ratio (GSH/GSSG), interleukin-1beta (IL-1β), and 8-isoprostane (8-iso-PGF_2_α) (13–15). Oxidative stress and mitochondrial dysfunction contribute to T2DM progression and influence cardiac electrophysiology through ion channel remodeling, autonomic instability, and myocardial energetics (16–18). This can cause QTc prolongation, QTd variability, and QRS-axis deviations of the ECG. These features of the ECG can be used as a non-invasive surrogate measure of systemic metabolic load and inflammation to assess disease progression and cardiovascular risk in T2DM patients.

Control of T2DM requires control of blood pressure and cardiovascular comorbidities to reduce the risk of T2DM progression through appropriate medication use, diet, and exercise (19). Medication use is also indicated for subclinical cardiac pathology including raised blood pressure, and diabetes (20). Multifactorial treatment options need consider obesity, inflammation, insulin resistance, oxidative stress, dyslipidemia, coagulopathy, and endothelial dysfunction as well as comprehensive multifactorial interventions in newly diagnosed T2DM associated with cardiac pathology (21). Metformin, thiazolidinediones, and novel anti-diabetic drugs, such as glucagon-like peptide-1 and sodium-glucose linked transporter-2 inhibitors, are also currently prescribed to improve insulin resistance (22, 23).

Hypertension and diabetes are interconnected and can lead to 50% faster chronic micro- and macro-vascular problems than in patients with no hypertension (24). Hyperglycemia can cause hypertension directly through microaneurysms, cytokines, and inflammogenic effects, and by several enzymes, osmotic agents, and vasoconstrictors (25). Hyperglycemia and arterial hypertension aggravate cardiovascular conditions by increasing the risk of left ventricular hypertrophy, coronary and cerebrovascular cardiac and brain lesions, resulting in a worse prognosis (26, 27). High blood pressure, rapid occurrence of complications, poorer prognosis, and increased cardiovascular morbidity and mortality are associated with diabetes and arterial hypertension (27). Some recommendations and protocols seek to achieve normotension or good arterial hypertension control in diabetics and those with impaired fasting glucose (28). Better arterial blood pressure regulation reduces erythrocytic hyperviscosity, arterial stiffness, cardiac hypertrophy with ventricular dysfunctions, glomerular blood clots, urine albumin, and other glucometabolic markers (29, 30).

Research has shown that ECG biomarkers can help track disease progression in diabetes, hypertension, and cardiovascular diseases and predict major adverse cardiac events (31, 32). Recently, biomedical engineering and bioengineering have relied on ECG indicators beyond clinical contexts. Continuous ECG monitoring with smart wearables allows real-time cardiac anomaly diagnosis and risk classification, enabling tailored medication. These developments enable adaptive algorithms to use patient-specific data for personalized feedback and treatment. These breakthroughs support precision health, digital therapies, and multimodal sensing, combining engineering with patient-centered care (33, 34). The advantage of ECG is its noninvasive nature, ease of interpretation, availability, and low cost compared to metabolic biomarkers (35). In addition, ECG findings have been linked the metabolic syndrome components, such as hypertension and dyslipidemia, and cardiac pathology (36, 37). Identifying specific biomarkers that link these diseases has been extended to community screening, particularly in rural populations, where they offer a non-invasive and efficient means of identifying asymptomatic cardiac conditions (38, 39). Moreover, the integration of ECG data with artificial intelligence (AI) has enhanced the diagnostic potential, enabling the early detection of subclinical diabetes complications that provides a cost-effective and accessible diagnostic approach (40, 41). Screening of cardiovascular disease progression with ECG has also shown that several ECG biomarkers are specific for monitoring progression of diabetes and hypertension in addition to CVD. These biomarkers include PQ interval, QRS complex, and QT/QTc (42). Due to the intricate relationship between diabetes and comorbidities such as hypertension and cardiovascular disease, classifying T2DM patients by comorbidities and treatment regimens including medication use is difficult. The current study examined how antilipid, antiplatelet, antihypertensive, and antidepressant medications affect ECG biomarkers in individuals with type 2 diabetes. It compared patient classification model results generated without considering diabetes medication status to assess the added value of including ECG markers. Furthermore, the study evaluated how these non-diabetes medications influence ECG biomarkers across different T2DM stages and comorbidity groups in a screening context, as well as their impact on the accuracy of classifying diabetes progression.

## 2 Materials and methods

### 2.1 Recruitment of participants

Data from 581 individuals participating in the Diabetes Health Screening Clinic (DiabHealth) at Charles Sturt University, Albury, Australia, were included in the analysis of blood and urine specimens. The study obtained ethical approval from the University Human Ethics Committee under Protocol Number 2006-042, and all participants provided written informed consent, including agreement for the publication of results. A control group with a screening glucose of <5.6 mmol/L, a prediabetes group with a screening glucose of 5.6–6.9 mmol/L, and a type 2 diabetes group with screening glucose ≥7 mm/L were compared. No exclusion criteria were established for this study, as patients participated in a public screening clinic focused on diabetes progression and diabetes complications. Gender, age, and the use of medications, including antiplatelet agents (AntiPL), lipid-lowering agents (AntiLP), antihypertensive medications (HT-med), and antidepressants, were collected for all patients in the study. The ECGs were recorded using a GE Healthcare MAC™ 5500 HD 12-lead electrocardiograph to obtain PQ interval conduction time, QRS duration, QTc interval, and QT dispersion (QTd). These metrics were analyzed from 10-s and 5-min recordings. These intervals represent important electrophysiological mechanisms related to diabetic pathogenesis and cardiovascular consequences. Autonomic dysfunction and decreased parasympathetic regulation in diabetes can affect atrioventricular conduction time, which affects the PQ interval (43, 44). QRS duration measures intraventricular conduction, and small delays may indicate structural or electrical changes like left ventricular hypertrophy or fibrosis (45, 46). The QTc interval and QTd measure ventricular repolarization heterogeneity, which predicts arrhythmic risk and sudden cardiac mortality, especially in diabetics (47–49). In diabetes, extended QTc and elevated QTd are linked to insulin resistance, glycemic management, and cardiovascular mortality (50, 51). These ECG indicators enable a comprehensive assessment of conduction, depolarization, and repolarization issues associated with diabetes and cardiovascular risk. The study focused on ECG biomarkers and diabetes progression, particularly in the context of comorbidities and medication use, over a 10-year period, utilizing data exclusively from first-admission records.

### 2.2 Statistical analysis and machine learning

Microsoft Excel (Office 365, Microsoft) was used to analyze descriptive data, and the results are presented as mean ± standard deviation (x ± SD). The machine learning process and statistical analyses were created to determine the significance of characteristics in predicting diabetes status and to find significant group differences. The Synthetic Minority Oversampling Technique (SMOTE), which was used to address class imbalance in the dataset, ensured that the four clinical subgroups—no comorbidities, hypertension, cardiovascular disease, and combined hypertension and cardiovascular disease—all had balanced representation of the no diabetes, prediabetes, and type 2 diabetes mellitus (T2DM) groups (52). The Support Vector Machine (SVM) consistently produced the greatest classification accuracy for differentiating diabetes groups among the machine learning models examined, including logistic regression, random forests, and k-nearest neighbors. It was chosen as the main classifier due to its performance and ability to handle high-dimensional data. SVM maximizes the gap between the closest support vectors (data points closest to the border) by finding the best hyperplane in the feature space that separates data points of various classes (53). This maximum improves the model’s generalizability and reduces the possibility of misclassification. By adjusting the regularization parameter (C), kernel type, and gamma settings, a grid search and 5-fold cross-validation were used to improve the SVM hyperparameters. As a measure of model stability, the associated standard deviation was provided, and the model with the greatest mean cross-validated accuracy was chosen as the best-performing model. The contribution of each feature was then measured by applying permutation significance to the optimum SVM model. This helped determine the most significant predictors by rearranging the values of each characteristic and calculating the change in model accuracy that resulted from these changes. Bar plots were used to display the results, and feature names were reformatted for readability. Pandas, scikit-learn, and imbalanced-learn were used in Python for further statistical studies. Kruskal–Wallis tests for non-parametric group differences were used to evaluate continuous data, whereas Chi-square tests using contingency tables were used to investigate categorical variables (54). To control for the increased risk of Type I errors due to multiple comparisons, we applied the Bonferroni correction by adjusting the significance threshold (*α*) from the conventional 0.05 level to *α/m* to include the total number of pairwise tests conducted (55). Significant variables were further investigated using paired Mann–Whitney U tests (56). This integrated methodology provides a comprehensive evaluation of the primary parameters that influence diabetes categorization and development by combining machine learning with robust statistical methodologies.

## 3 Results

### 3.1 Demographics and statistical analysis

Data from five hundred and eighty-one participants were included in the analysis.

Table 1 summarizes the demographic and clinical characteristics of the Healthy, Prediabetes, and T2DM participant groups, including pairwise *post-hoc* Chi-square tests with the Bonferroni correction for categorical variables. Prescription of statins (AntiLP-MedUse) was significantly higher in the T2DM group, with 33.3% of individuals compared to the Healthy and Prediabetes groups (*p* < 0.001, Kruskal–Wallis). *Post-hoc* pairwise Chi-square tests with Bonferroni correction revealed a significant difference between the Healthy and T2DM groups (*p* < 0.001). Medications, such as antidepressant medications, did not vary significantly between groups (*p* = 0.383). The mean age of 55.22 years in the Healthy group to 58.92 years in the T2DM group was not significant. Similarly, gender distribution was predominantly female in all groups but was not significantly different between the three groups. ECG features, however, reveal a significant difference in QTd-10 s, suggesting a potential link to diabetes. Pairwise comparisons also highlight that QTd-5 min is significantly different between the Healthy and T2DM groups.

**Table 1:** Demographics, Kruskal–Wallis, and Mann–Whitney analysis of the population without comorbidities.

Feature	Healthy (H)	Prediabetes (P)	T2DM (T)	Kruskal–Wallis/*χ*^2^ *p*-value	Mann–Whitney/Bonferroni		
					*p*-value (H vs P)	*p*-value (H vs T)	*p*-value (P vs T)
*N*	123	21	12	–	–	–	–
AntiLP-MedUse	**No: 121 (98.4%)**	**No: 21 (100.0%)**	**No: 8 (66.7%)**	**<0.001**	1.000	**<0.001**	**0.023**
	**Yes: 2 (1.6%)**	**Yes: 0 (0.0%)**	**Yes: 4 (33.3%)**				
AntiPL-MedUse	No: 123 (100.0%)	No: 21 (100.0%)	No: 12 (100.0%)	1.000	1.000	1.000	1.000
	Yes: 0 (0.0%)	Yes: 0 (0.0%)	Yes: 0 (0.0%)				
Birth Age [yrs]	55.22 ± 11.60	57.76 ± 9.55	58.92 ± 8.70	0.296	0.309	0.199	0.694
DM-MedUse	**No: 123 (100.0%)**	**No: 21 (100.0%)**	**No: 12 (100.0%)**	**<0.001**	1.000	**<0.001**	**<0.001**
	**Yes: 0 (0.0%)**	**Yes: 0 (0.0%)**	**Yes: 0 (0.0%)**				
Gender	F: 75 (61.0%)	F: 10 (47.6%)	F: 8 (66.7%)	0.450	0.363	0.939	0.488
	M: 48 (39.0%)	M: 11 (52.4%)	M: 4 (33.3%)				
Grade-10 s	1.18 ± 0.53	1.19 ± 0.51	1.33 ± 0.65	0.436	0.746	0.202	0.469
Grade-5 min	1.10 ± 0.37	1.24 ± 0.62	1.17 ± 0.58	0.538	0.267	0.865	0.668
HT-MedUse	No: 123 (100.0%)	No: 21 (100.0%)	No: 12 (100.0%)	1.000	1.000	1.000	1.000
	Yes: 0 (0.0%)	Yes: 0 (0.0%)	Yes: 0 (0.0%)				
Other meds depression	No: 115 (93.5%)	No: 20 (95.2%)	No: 10 (83.3%)	0.383	1.000	0.480	0.607
	Yes: 8 (6.5%)	Yes: 1 (4.8%)	Yes: 2 (16.7%)				
PQ-10 s [ms]	168.70 ± 20.83	176.19 ± 26.54	165.25 ± 17.22	0.667	0.397	0.892	0.409
PQ-5 min [ms]	169.15 ± 24.01	166.95 ± 16.11	167.75 ± 29.05	0.982	0.973	0.844	0.955
QRS-10 s [ms]	95.81 ± 6.70	97.38 ± 5.21	97.50 ± 8.50	0.455	0.255	0.513	0.954
QRS-5 min [ms]	95.13 ± 10.20	96.38 ± 6.20	97.92 ± 7.06	0.195	0.193	0.166	0.735
QRS-Axis-10 s [^o^]	32.07 ± 24.70	25.90 ± 26.39	21.58 ± 25.99	0.235	0.256	0.167	0.680
QRS-Axis-5 min [^o^]	**24.08 ± 22.48**	25.81 ± 24.37	**38.00 ± 23.38**	0.101	0.743	**0.035**	0.088
QTc-10 s [ms]	423.21 ± 15.93	**429.76 ± 19.46**	**416.00 ± 6.45**	0.067	0.170	0.089	**0.023**
QTc-5 min [ms]	424.72 ± 18.07	**429.24 ± 14.27**	**420.67 ± 13.03**	0.118	0.091	0.372	**0.039**
QTd-10 s [ms]	**53.94 ± 17.99**	**70.76 ± 20.53**	59.67 ± 18.25	**<0.001**	**<0.001**	0.349	0.177
QTd-5 min [ms]	**58.25 ± 15.01**	**66.62 ± 14.97**	**67.33 ± 12.00**	**0.008**	**0.012**	**0.037**	0.612

Bold values indicate statistically significant differences (*p* < 0.05). A single bold pairwise *p*-value indicates significance between two classes only based on Mann–Whitney/Bonferroni analysis. AntiLP-MedUse, Antilipid medication use; AntiPL-MedUse, Antiplatelet medication use; DM-MedUse, Diabetes medication use; HT-MedUse, Hypertension medication use; PQ-10 s, PQ interval measured over 10 s; PQ-5 min, PQ interval measured over 5 min; QRS-10 s, QRS complex duration measured over 10 s; QRS-5 min, QRS complex duration measured over 5 min; QRS-Axis-10 s, QRS axis deviation measured over 10 s; QRS-Axis-5 min, QRS axis deviation measured over 5 min; QTc-10 s, Corrected QT interval measured over 10 s; QTc-5 min, Corrected QT interval measured over 5 min; QTd-10 s, QT dispersion measured over 10 s; QTd-5 min, QT dispersion measured over 5 min.

Demographic and clinical characteristics of the participants with hypertension are presented in Table 2, along with pairwise *post-hoc* Chi-square tests with the Bonferroni correction for categorical variables. In Table 2, the term Control refers to patients diagnosed with hypertension only. Medication is a key differentiator among the groups, with AntiLP-MedUse, as indicated by *post-hoc* pairwise Chi-square tests with Bonferroni correction, showing a significant difference between Healthy and T2DM, and between Prediabetes and T2DM. Diabetes medication was prescribed significantly more often for the hypertension groups compared to the T2DM group. As expected, increased hypertension-specific medication use (HT-MedUse) was observed in all three diabetes-associated groups compared to the Healthy group. ECG features reveal several significant distinctions. PQ-10 s differed significantly between groups, with a significant difference between the Healthy and Prediabetes groups. QTd-10 s and QTd-5 min also show significant differences, particularly between the Prediabetes and T2DM groups.

**Table 2:** Demographics, Kruskal–Wallis, and Mann–Whitney analysis of the population with hypertension.

Feature	Control (C)	Prediabetes (P)	T2DM (T)	Kruskal–Wallis/*χ*^2^ *p*-value	Mann–Whitney/Bonferroni		
					*p*-value (C vs P)	*p*-value (C vs T)	*p*-value (P vs T)
*N*	80	20	48	–	–	–	–
AntiLP-Med Use	**No: 68 (85.0%)**	No: 14 (70.0%)	**No: 23 (47.9%)**	**<0.001**	0.216	**<0.001**	0.162
	**Yes: 12 (15.0%)**	Yes: 6 (30.0%)	**Yes: 25 (52.1%)**				
AntiPL-Med Use	No: 80 (100.0%)	No: 20 (100.0%)	No: 48 (100.0%)	1.000	1.000	1.000	1.000
	Yes: 0 (0.0%)	Yes: 0 (0.0%)	Yes: 0 (0.0%)				
Birth Age [yrs]	65.88 ± 11.10	No: 68.35 ± 10.12	No: 63.81 ± 9.74	0.168	0.413	0.176	0.071
DM-Med Use	**No: 80 (100.0%)**	**No: 19 (95.0%)**	**No: 11 (22.9%)**	**<0.001**	0.451	**<0.001**	<**0.001**
	**Yes: 0 (0.0%)**	**Yes: 1 (5.0%)**	**Yes: 37 (77.1%)**				
Gender	F: 44 (55.0%)	F: 10 (50.0%)	F: 24 (50.0%)	0.832	0.880	0.714	1.000
	M: 36 (45.0%)	M: 10 (50.0%)	M: 24 (50.0%)				
Grade-10 s	1.26 ± 0.57	1.05 ± 0.22	1.25 ± 0.56	0.270	0.107	0.875	0.143
Grade-5 min	**1.21 ± 0.50**	1.00 ± 0.00	**1.12 ± 0.39**	0.095	**0.046**	0.278	0.141
HT-Med Use	**No: 45 (56.2%)**	No: 6 (30.0%)	**No: 12 (25.0%)**	**<0.001**	0.064	**0.001**	0.901
	**Yes: 35 (43.8%)**	Yes: 14 (70.0%)	**Yes: 36 (75.0%)**				
Other meds depression	No: 70 (87.5%)	No: 17 (85.0%)	No: 39 (81.2%)	0.629	1.000	0.480	0.984
	Yes: 10 (12.5%)	Yes: 3 (15.0%)	Yes: 9 (18.8%)				
PQ-10 s [ms]	**169.40 ± 22.02**	**183.30 ± 19.01**	**170.96 ± 23.12**	**0.037**	0.**008**	0.788	**0.043**
PQ-5 min [ms]	169.91 ± 23.25	171.95 ± 24.35	166.08 ± 25.99	0.562	0.682	0.406	0.335
QRS-10 s [ms]	104.59 ± 17.77	104.70 ± 9.73	106.44 ± 17.15	0.354	0.188	0.327	0.695
QRS-5 min [ms]	**101.64 ± 16.05**	101.80 ± 12.91	**103.58 ± 8.65**	0.081	0.528	**0.021**	0.557
QRS-Axis-10 s [^o^]	**7.42 ± 28.01**	2.50 ± 26.24	**20.27 ± 32.19**	**0.047**	0.325	**0.035**	0.059
QRS-Axis-5 min [^o^]	15.62 ± 29.24	**5.95 ± 32.52**	**24.71 ± 27.14**	0.055	0.231	0.079	**0.032**
QTc-10 s [ms]	427.88 ± 20.50	429.60 ± 18.93	426.31 ± 18.31	0.501	0.430	0.528	0.255
QTc-5 min [ms]	**431.81 ± 20.26**	428.65 ± 17.11	**424.73 ± 16.74**	0.090	0.976	**0.037**	0.139
QTd-10 s [ms]	**74.84 ± 38.84**	**86.25 ± 43.58**	**51.00 ± 14.33**	**<0.001**	0.259	**<0.001**	**<0.001**
QTd-5 min [ms]	**81.85 ± 37.64**	**83.35 ± 34.58**	**58.62 ± 9.84**	**0.001**	0.743	**0.001**	**0.002**

Bold values indicate statistically significant differences (*p* < 0.05). Bonferroni correction and pairwise *p*-value shown in bold indicate significance between two classes only based on Mann–Whitney/Bonferroni analysis. AntiLP-MedUse, Antilipid medication use; AntiPL-MedUse, Antiplatelet medication use; DM-MedUse, Diabetes medication use; HT-MedUse, Hypertension medication use; PQ-10 s, PQ interval measured over 10 s; PQ-5 min, PQ interval measured over 5 min; QRS-10 s, QRS complex duration measured over 10 s; QRS-5 min, QRS complex duration measured over 5 min; QRS-Axis-10 s, QRS axis deviation measured over 10 s; QRS-Axis-5 min, QRS axis deviation measured over 5 min; QTc-10 s, Corrected QT interval measured over 10 s; QTc-5 min, Corrected QT interval measured over 5 min; QTd-10 s, QT dispersion measured over 10 s; QTd-5 min, QT dispersion measured over 5 min.

AntiLP-MedUse in the cardiovascular disease cohort across the three groups was significantly more common in the T2DM group (Table 3). *Post-hoc* pairwise Chi-square tests with the Bonferroni correction revealed significant differences between the Control and T2DM groups, as well as between the Prediabetes and T2DM groups. In Table 3, the term Control refers to patients diagnosed with cardiovascular disease only. Diabetes medication was only prescribed in the T2DM group. In line with previous results, gender distribution revealed significant variation, with a higher proportion of males in the prediabetes group compared to the control and T2DM groups. *Post-hoc* pairwise comparisons revealed a significant difference between the Control and Prediabetes groups, but not between Control and T2DM groups, or between Prediabetes and T2DM groups. ECG features show mixed results. PQ-10 s exhibited a significant difference between the prediabetes and T2DM groups. QTc-5 min values were significantly different among the groups, particularly between the control, prediabetes, and T2DM groups, with the longest intervals observed in prediabetes (430.23 ms), which is still within the normal range for both men and women.

**Table 3:** Demographics, Kruskal–Wallis, and Mann–Whitney analysis of the population with cardiovascular disease.

Feature	Control (C)	Prediabetes (P)	T2DM (T)	Kruskal–Wallis/*χ*^2^ *p*-value	Mann–Whitney/Bonferroni		
					*p*-value (C vs P)	*p*-value (C vs T)	*p*-value (P vs T)
*N*	34	13	14	–	–	–	–
AntiLP-Med Use	**No: 29 (85.3%)**	**No: 12 (92.3%)**	**No: 6 (42.9%)**	**0.002**	0.876	**0.008**	**0.021**
	**Yes: 5 (14.7%)**	**Yes: 1 (7.7%)**	**Yes: 8 (57.1%)**				
AntiPL-Med Use	No: 10 (29.4%)	No: 6 (46.2%)	No: 2 (14.3%)	0.193	0.460	0.463	0.164
	Yes: 24 (70.6%)	Yes: 7 (53.8%)	Yes: 12 (85.7%)				
Birth Age [yrs]	68.12 ± 11.31	67.69 ± 11.61	65.07 ± 10.70	0.607	0.821	0.296	0.680
DM-Med Use	**No: 34 (100.0%)**	**No: 13 (100.0%)**	**No: 3 (21.4%)**	**0**	1.000	**<0.001**	**<0.001**
	**Yes: 0 (0.0%)**	**Yes: 0 (0.0%)**	**Yes: 11 (78.6%)**				
Gender	**F: 26 (76.5%)**	F: 4 (30.8%)	**F: 6 (42.9%)**	**0.006**	**0.010**	0.056	0.802
	**M: 8 (23.5%)**	M: 9 (69.2%)	**M: 8 (57.1%)**				
Grade-10 s	1.44 ± 0.79	1.38 ± 0.77	1.21 ± 0.58	0.641	0.827	0.354	0.566
Grade-5 min	1.56 ± 0.82	1.31 ± 0.63	1.14 ± 0.53	0.142	0.370	0.062	0.307
HT-Med Use	No: 34 (100.0%)	No: 13 (100.0%)	No: 14 (100.0%)	1	1	1	1
	Yes: 0 (0.0%)	Yes: 0 (0.0%)	Yes: 0 (0.0%)				
Other meds depression	No: 31 (91.2%)	No: 13 (100.0%)	No: 12 (85.7%)	0.393	0.660	0.965	0.496
	Yes: 3 (8.8%)	Yes: 0 (0.0%)	Yes: 2 (14.3%)				
PQ-10 s [ms]	163.56 ± 20.22	**168.15 ± 15.40**	**156.50 ± 18.35**	0.063	0.354	0.083	**0.019**
PQ-5 min [ms]	178.50 ± 33.00	171.77 ± 18.84	164.00 ± 14.09	0.262	0.617	0.123	0.262
QRS-10 s [ms]	97.79 ± 9.43	102.08 ± 9.63	94.29 ± 12.11	0.149	0.158	0.285	0.086
QRS-5 min [ms]	98.88 ± 8.95	106.00 ± 18.41	99.29 ± 4.23	0.468	0.322	0.973	0.184
QRS-Axis-10 s [^o^]	11.29 ± 24.90	21.38 ± 33.61	22.71 ± 16.27	0.367	0.528	0.148	0.785
QRS-Axis-5 min [^o^]	**17.85 ± 19.04**	24.31 ± 34.68	**34.07 ± 7.75**	**0.014**	0.766	**0.002**	0.161
QTc-10 s [ms]	422.59 ± 18.12	432.62 ± 22.95	429.79 ± 9.36	0.189	0.186	0.116	0.845
QTc-5 min [ms]	**424.97 ± 16.44**	**430.23 ± 18.93**	**412.86 ± 18.69**	**0.048**	0.329	**0.038**	**0.041**
QTd-10 s [ms]	60.50 ± 19.80	62.85 ± 33.75	56.14 ± 29.60	0.436	0.729	0.203	0.465
QTd-5 min [ms]	45.09 ± 9.79	47.15 ± 9.99	55.07 ± 34.41	0.771	0.431	0.820	0.903

Bold values indicate statistically significant differences (*p* < 0.05). A single bold pairwise *p*-value indicates significance between two classes only based on Mann–Whitney/Bonferroni analysis. AntiLP-MedUse, Antilipid medication use; AntiPL-MedUse, Antiplatelet medication use; DM-MedUse, Diabetes medication use; HT-MedUse, Hypertension medication use; PQ-10 s, PQ interval measured over 10 s; PQ-5 min, PQ interval measured over 5 min; QRS-10 s, QRS complex duration measured over 10 s; QRS-5 min, QRS complex duration measured over 5 min; QRS-Axis-10 s, QRS axis deviation measured over 10 s; QRS-Axis-5 min, QRS axis deviation measured over 5 min; QTc-10 s, Corrected QT interval measured over 10 s; QTc-5 min, Corrected QT interval measured over 5 min; QTd-10 s, QT dispersion measured over 10 s; QTd-5 min, QT dispersion measured over 5 min.

ECG biomarkers and medication were able to separate Control, Prediabetes, and T2DM individuals with hypertension (HT) and cardiovascular disease (CVD) (Table 4). In Table 4, the term Control refers to patients diagnosed with both hypertension and cardiovascular diseases. AntiLP-medication use differed significantly amongst diabetes groups, emphasizing its importance in diabetes classification models. *Post-hoc* comparisons revealed significant differences between the Control and T2DM groups (*p* < 0.001) and the Prediabetes and T2DM groups (*p* = 0.005). Previous research has shown that antidiabetic therapy affects disease progression and cardiovascular risk assessment and indicates the importance of using diabetes medication in the model (57). Hypertension medication used in the model may indirectly indicate diabetes progression. T2DM patients also reported higher use of antidepressants compared to Control and Prediabetes (*p* = 0.010). Since antidepressants have been connected to metabolic abnormalities and diabetes risk, this finding shows a link between depression and diabetes progression that merits additional study.

**Table 4:** Demographics, Kruskal–Wallis, and Mann–Whitney analysis of the population with both hypertension and cardiovascular diseases.

Feature	Control (C)	Prediabetes (P)	T2DM (T)	Kruskal–Wallis/χ^2^ *p*-value	Mann–Whitney/Bonferroni		
					*p*-value (C vs P)	*p*-value (C vs T)	*p*-value (P vs T)
*N*	95	26	95	–	–	–	–
AntiLP-Med Use	**No: 56 (58.9%)**	**No: 17 (65.4%)**	**No:31 (32.6%)**	**<0.001**	0.713	<**0.001**	**0.005**
	**Yes: 39 (41.1%)**	**Yes: 9 (34.6%)**	**Yes: 64 (67.4%)**				
AntiPL-Med Use	No: 25 (26.3%)	No: 5 (19.2%)	No: 17 (17.9%)	0.352	0.628	0.221	1.000
	Yes: 70 (73.7%)	Yes: 21 (80.8%)	Yes: 78 (82.1%)				
Birth Age [yrs]	70.37 ± 8.88	69.96 ± 8.40	69.77 ± 8.49	0.764	0.654	0.488	0.990
DM-Med Use	**No: 95 (100.0%)**	**No: 26 (100.0%)**	**No: 26 (27.4%)**	**<0.001**	1.000	<**0.001**	<**0.001**
	**Yes: 0 (0.0%)**	**Yes: 0 (0.0%)**	**Yes: 69 (72.6%)**				
Gender	F: 60 (63.2%)	F: 12 (46.2%)	F: 46 (27.4%)	0.081	0.180	0.058	1.000
	M: 35 (36.8%)	M: 14 (53.8%)	M: 49 (72.6%)				
Grade-10 s	**1.41 ± 0.69**	**1.12 ± 0.43**	**1.45 ± 0.68**	**0.036**	**0.027**	0.531	**0.009**
Grade-5 min	1.60 ± 0.74	1.54 ± 0.76	1.56 ± 0.71	0.867	0.633	0.715	0.800
HT-Med Use	**No: 27 (28.4%)**	**No: 7 (26.9%)**	**No: 6 (6.3%)**	**<0.001**	1.000	<**0.001**	**0.008**
	**Yes: 68 (71.6%)**	**Yes: 19 (73.1%)**	**Yes: 89 (93.7%)**				
Other meds depression	**No: 87 (91.6%)**	No: 24 (92.3%)	**No: 73 (76.8%)**	**0.010**	1.000	**0.010**	0.140
	**Yes: 8 (8.4%)**	Yes: 2 (7.7%)	**Yes: 22 (23.2%)**				
PQ-10 s [ms]	181.69 ± 36.73	176.27 ± 32.79	173.40 ± 21.63	0.221	0.512	0.075	0.892
PQ-5 min [ms]	182.20 ± 28.39	177.00 ± 22.74	178.29 ± 22.78	0.590	0.348	0.442	0.791
QRS-10 s [ms]	113.15 ± 26.29	113.15 ± 26.74	105.65 ± 15.51	0.875	0.892	0.638	0.730
QRS-5 min [ms]	104.53 ± 14.24	101.69 ± 14.22	101.69 ± 15.35	0.391	0.332	0.231	0.688
QRS-Axis-10 s [^o^]	**−5.33 ± 45.15**	−4.23 ± 41.38	**8.19 ± 31.06**	**0.018**	0.879	**0.006**	0.146
QRS-Axis-5 min [^o^]	3.84 ± 29.93	9.58 ± 32.22	7.81 ± 25.18	0.355	0.441	0.161	0.791
QTc-10 s [ms]	**437.86 ± 17.55**	435.00 ± 21.72	**430.68 ± 24.84**	**0.001**	0.441	**<0.001**	0.126
QTc-5 min [ms]	431.45 ± 24.23	428.69 ± 14.84	426.02 ± 19.40	0.147	0.806	0.068	0.214
QTd-10 s [ms]	**77.11 ± 32.31**	**83.46 ± 35.53**	**71.63 ± 33.67**	**0.038**	0.326	**0.037**	**0.047**
QTd-5 min [ms]	60.47 ± 19.63	65.08 ± 30.38	66.26 ± 32.73	0.848	0.975	0.586	0.733

Bold values indicate statistically significant differences (*p* < 0.05). A single bold pairwise *p*-value indicates significance between two classes only based on Mann–Whitney/Bonferroni analysis. AntiLP-MedUse, Antilipid medication use; AntiPL-MedUse, Antiplatelet medication use; DM-MedUse, Diabetes medication use; HT-MedUse, Hypertension medication use; PQ-10 s, PQ interval measured over 10 s; PQ-5 min, PQ interval measured over 5 min; QRS-10 s, QRS complex duration measured over 10 s; QRS-5 min, QRS complex duration measured over 5 min; QRS-Axis-10 s, QRS axis deviation measured over 10 s; QRS-Axis-5 min, QRS axis deviation measured over 5 min; QTc-10 s, Corrected QT interval measured over 10 s; QTc-5 min, Corrected QT interval measured over 5 min; QTd-10 s, QT dispersion measured over 10 s; QTd-5 min, QT dispersion measured over 5 min.

Several ECG biomarkers showed statistically significant differences, supporting their use in machine learning-based diabetes classification models. QRS-Axis-10 s was significantly different between Control vs. T2DM (*p* = 0.006), suggesting that diabetic individuals with HT and CVD may have more structural heart or electrical remodeling issues. T2DM patients had longer grade-10 s (*p* = 0.036) conduction delays, which may indicate autonomic dysfunction and diabetes-related conduction anomalies (58). QTc-10 s (*p* = 0.001) and QTd-10 s (*p* = 0.038) demonstrated substantial differences between the three groups, with T2DM patients having wider repolarization times, a risk factor for arrhythmias and sudden cardiac death, specifically between Control and T2DM (*p* < 0.001) (59).

### 3.2 ECG-based classification and feature selection

**Figure 1:**
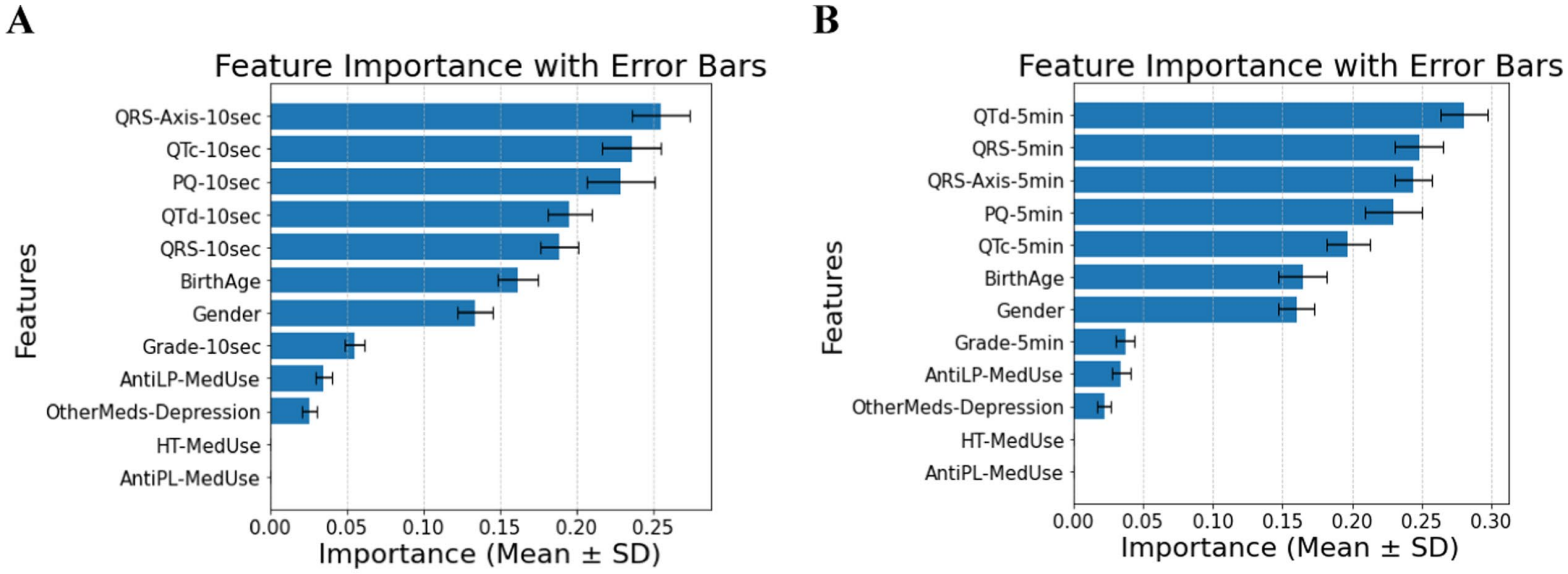
**(A)** Feature importance for patients without comorbidities, for ECG biomarkers measured for 10 s. **(B)** Feature importance for patients without comorbidities, for ECG biomarkers measured 5 min. AntiLP-MedUse, Antilipid medication use; AntiPL-MedUse, Antiplatelet medication use; HT-MedUse, Hypertension medication use; PQ-10 s, PQ interval measured over 10 s; PQ-5 min, PQ interval measured over 5 min; QRS-10 s, QRS complex duration measured over 10 s; QRS-5 min, QRS complex duration measured over 5 min; QRS-Axis-10 s, QRS axis deviation measured over 10 s; QRS-Axis-5 min, QRS axis deviation measured over 5 min; QTc-10 s, Corrected QT interval measured over 10 s; QTc-5 min, Corrected QT interval measured over 5 min; QTd-10 s, QT dispersion measured over 10 s; QTd-5 min, QT dispersion measured over 5 min.

The feature relevance rankings for diabetes classification in patients without comorbidities using ECG biomarkers over 10 s (Panel A) and 5 min (Panel B) are shown in Figure 1. As the T2DM medication feature is excluded, QRS-Axis-10 s, QTc-10 s, PQ-10 s, and QTd-10 s for 10-s measures and QTd-5 min, QRS-5 min, QRS-Axis-5 min, and PQ-5 min for 5-min measurements are the most influential predictors. Medication-related parameters, especially anti-lipid and AntiLP-MedUse medication use, contribute to classification but are less important.

Combining demographic, clinical, and ECG variables reveals notable differences among the Healthy, Prediabetes, and T2DM groups, as summarized in Table 1. Classification accuracy was 0.86 ± 0.02 for 10-s intervals and 0.88 ± 0.02 for 5-min intervals, with a focus on cardiac ECG biomarkers. ECG biomarkers. Anti-lipid medication use reported by the Healthy and T2DM groups vary significantly. Among ECG biomarkers, QTd-10 s and QTd-5 min show significant differences between the Healthy and Prediabetes groups and between the Healthy and T2DM groups, respectively, as confirmed by pairwise comparisons. In contrast, anti-depression medication does not substantially vary, suggesting that antidepressant use is unrelated to diabetes status in this cohort.

**Figure 2:**
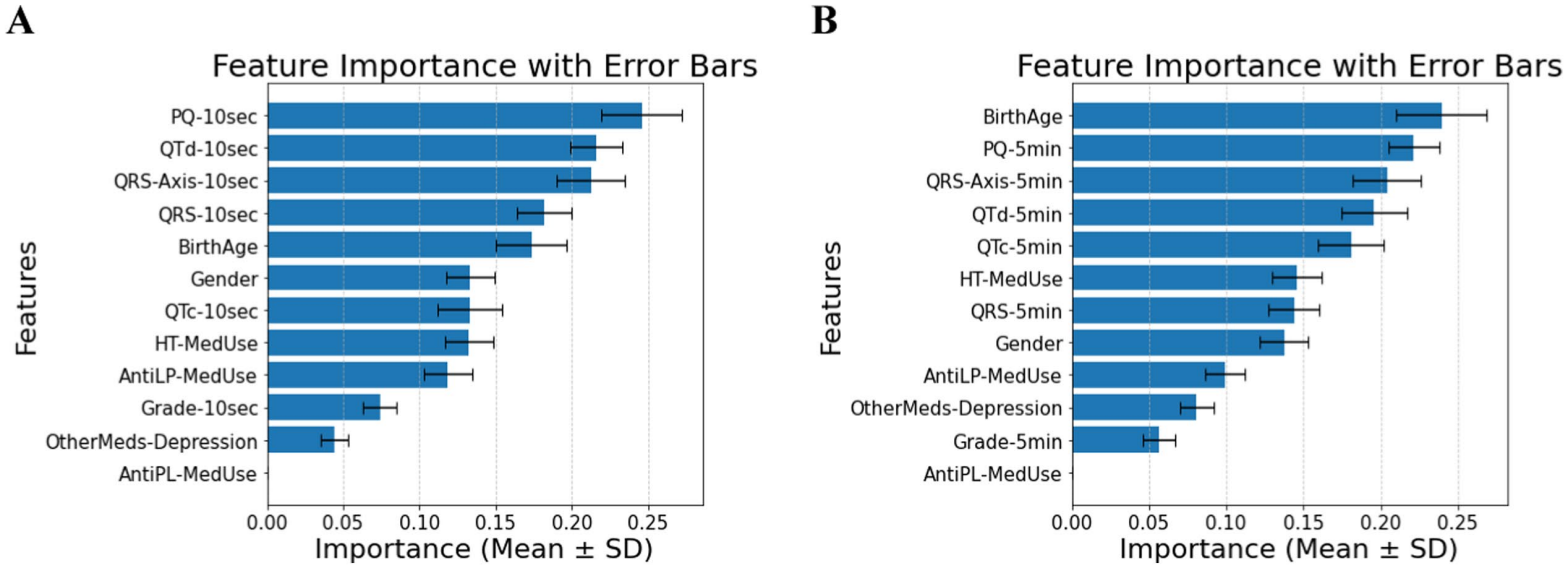
**(A)** Feature importance for patients with hypertension, for ECG biomarkers measured for 10 s. **(B)** Feature importance for patients with hypertension, for ECG biomarkers measured for 5 min. AntiLP-MedUse, Antilipid medication use; AntiPL-MedUse, Antiplatelet medication use; HT-MedUse, Hypertension medication use; PQ-10 s, PQ interval measured over 10 s; PQ-5 min, PQ interval measured over 5 min; QRS-10 s, QRS complex duration measured over 10 s; QRS-5 min, QRS complex duration measured over 5 min; QRS-Axis-10 s, QRS axis deviation measured over 10 s; QRS-Axis-5 min, QRS axis deviation measured over 5 min; QTc-10 s, Corrected QT interval measured over 10 s; QTc-5 min, Corrected QT interval measured over 5 min; QTd-10 s, QT dispersion measured over 10 s; QTd-5 min, QT dispersion measured over 5 min.

Many factors affect patients with diabetes and hypertension. Without the T2DM medication component, the predictive power shifts primarily to ECG biomarkers. In the 10-s measurement (Panel A of Figure 2), PQ-10 s, QTd-10 s, and QRS-Axis-10 s emerge as the dominant predictors, suggesting their strong role in capturing conduction and repolarization abnormalities. In contrast, the 5-min measurement (Panel B of Figure 2) highlights the most important features, including Birth Age, PQ-5 min, and QRS-Axis-5 min, suggesting that longer ECG recordings capture broader structural and conduction-related differences. The shift in feature rankings between Panels A and B highlights the influence of ECG biomarkers in predicting diabetes risk in hypertensive patients and underscores the role of measurement duration in feature importance distribution. Comparing demographic and clinical factors across the hypertensive diabetic groups shows a significant difference in the reported antilipid medication use between the Control and T2DM groups. Several ECG indicators further distinguish between the three groups. Significant variations in QTd-5 min and QTd-10 s exist between the Prediabetes and T2DM groups, and between the Control and T2DM groups. PQ-10 s difference is significant, with *post-hoc* comparisons confirming differences between Control and Prediabetes and Prediabetes vs. T2DM. QRS-Axis-10 s also distinguishes Control from T2DM. Demographic characteristics have a to minimal impact on diabetes classification in individuals with hypertension. As for QRS-Axis-5 min, although the overall group difference was marginal (*p* = 0.055), the difference between Prediabetes and T2DM was significant (*p* = 0.032), while the other pairwise comparisons were not. The results confirm that ECG biomarkers are key diabetes classification factors for hypertensive patients, with a classification accuracy of 0.72 ± 0.05 for the 10-s recording and 0.74 ± 0.08 for the 5-min recording.

**Figure 3:**
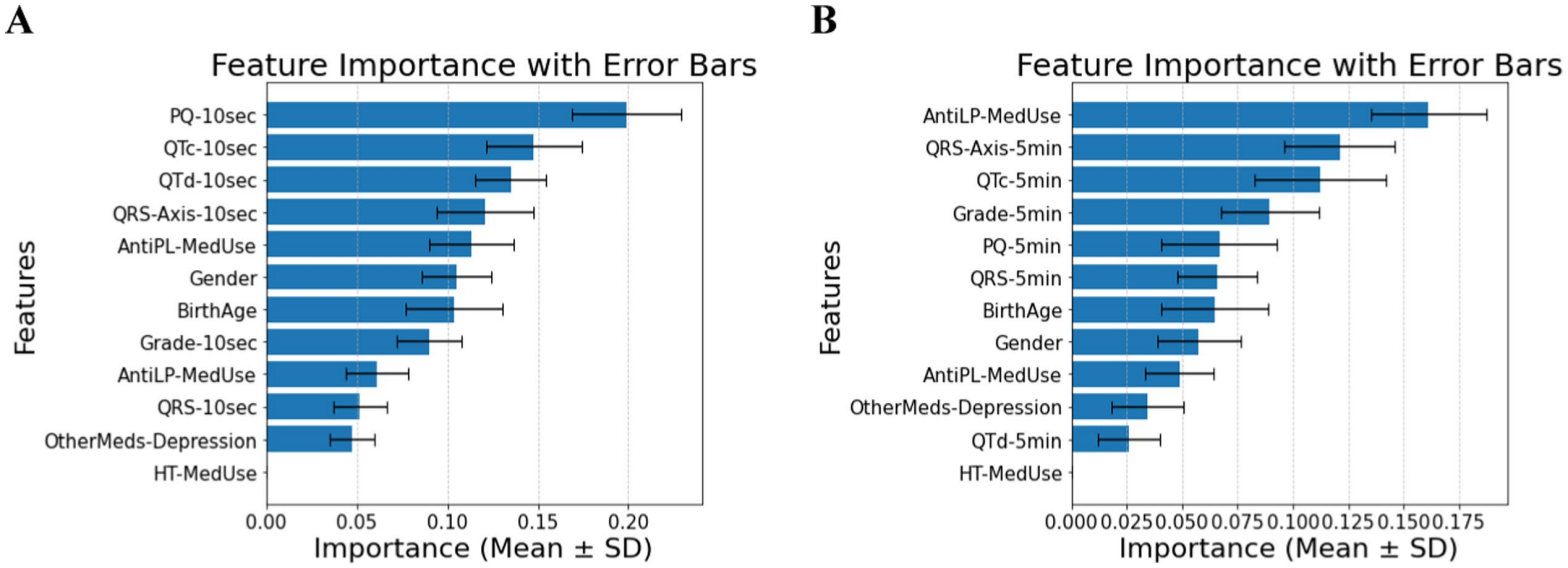
**(A)** Feature importance for patients with cardiovascular disease, for ECG biomarkers measured for 10 s. **(B)** Feature importance for patients cardiovascular disease, for ECG biomarkers measured for 5 min. AntiLP-MedUse, Antilipid medication use; AntiPL-MedUse, Antiplatelet medication use; HT-MedUse, Hypertension medication use; PQ-10 s, PQ interval measured over 10 s; PQ-5 min, PQ interval measured over 5 min; QRS-10 s, QRS complex duration measured over 10 s; QRS-5 min, QRS complex duration measured over 5 min; QRS-Axis-10 s, QRS axis deviation measured over 10 s; QRS-Axis-5 min, QRS axis deviation measured over 5 min; QTc-10 s, Corrected QT interval measured over 10 s; QTc-5 min, Corrected QT interval measured over 5 min; QTd-10 s, QT dispersion measured over 10 s; QTd-5 min, QT dispersion measured over 5 min.

In patients with cardiovascular disease, PQ-10 s, QTc-10 s, QTd-10 s, and QRS-Axis-10 s emerge as the most significant predictors, indicating that short-term conduction and repolarization measures gain prominence (Panel A of Figure 3). In the 5-min measurements, AntiLP-MedUse, QRS-Axis-5 min, and QTc-5 min dominate (Panel B of Figure 3). Notably, PQ-10 s showed marginal overall significance, but its pairwise comparison between Prediabetes and T2DM reached significance (*p* = 0.019). For the 5-min recordings, AntiLP-Med Use (*p* = 0.002), QRS-Axis-5 min (*p* = 0.014), and QTc-5 min (*p* = 0.048) showed the most substantial group differences, underscoring the dominance of long-term ECG measures and medication use in distinguishing between Control, Prediabetes, and T2DM groups (Panel B, Figure 3). These shifts emphasize the interplay between ECG biomarkers and medication use in cardiovascular disease patients, and the accuracies equal 0.74 ± 0.04 for the 10-s recording and 0.77 ± 0.07 for the 5-min recording. In addition to medication and ECG features being important in classification accuracy, gender indicated significant differences between the diabetes groups. However, the antidepressant medication used does not differ and contributes to the model, demonstrating that antidepressant use does not discriminate between cardiovascular disease patients with diabetes and patients with different comorbidities or no comorbidities.

**Figure 4:**
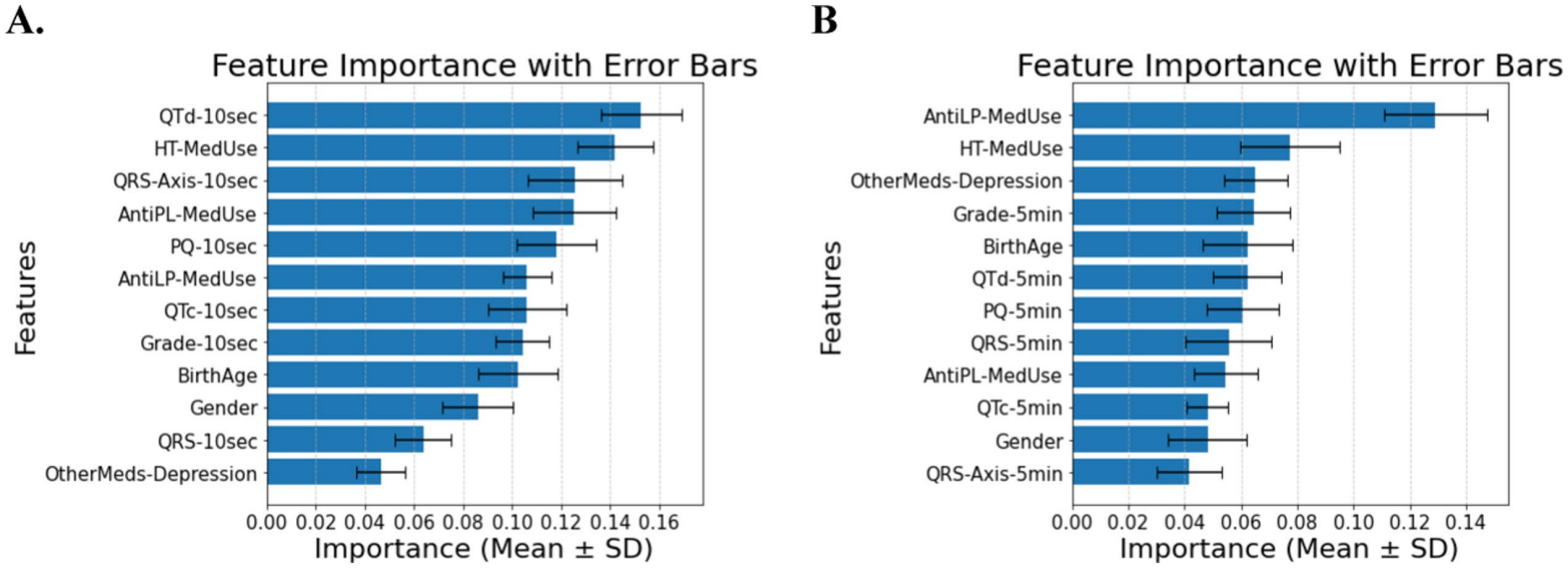
**(A)** Feature importance for patients with hypertension and cardiovascular disease, for ECG biomarkers measured for 10 s. **(B)** Feature importance for patients with hypertension and cardiovascular disease, for ECG biomarkers measured for 5 min. AntiLP-MedUse, Antilipid medication use; AntiPL-MedUse, Antiplatelet medication use; HT-MedUse, Hypertension medication use; PQ-10 s, PQ interval measured over 10 s; PQ-5 min, PQ interval measured over 5 min; QRS-10 s, QRS complex duration measured over 10 s; QRS-5 min, QRS complex duration measured over 5 min; QRS-Axis-10 s, QRS axis deviation measured over 10 s; QRS-Axis-5 min, QRS axis deviation measured over 5 min; QTc-10 s, Corrected QT interval measured over 10 s; QTc-5 min, Corrected QT interval measured over 5 min; QTd-10 s, QT dispersion measured over 10 s; QTd-5 min, QT dispersion measured over 5 min.

The classification of diabetes in individuals with hypertension and cardiovascular disease depends primarily on medication use, as expected, but includes ECG biomarkers, with minor contributions from demographic features. In Figure 4A (10-s analysis), QTd-10 s, HT-MedUse, and QRS-Axis-10 s emerge as the top predictors, highlighting the role of repolarization abnormalities and hypertension treatment. In Figure 4B (5-min analysis), AntiLP-MedUse, HT-MedUse, and OtherMeds-Depression dominate, suggesting that non-diabetes medications, particularly lipid-lowering and antidepressant treatments, influence long-term ECG variability. The classification accuracy for these models is 0.68 ± 0.06 in Figure 4A, and 0.64 ± 0.05 in Figure 4B, indicating performance loss. This shows that including provides a focus on physiological indicators. For individuals with hypertension and cardiovascular disease, Table 4 compares demographic, clinical, and ECG data from the Control, Prediabetes, and T2DM groups. HT-MedUse marks a significant difference, with 93.7% of T2DM patients using hypertension medication compared to the Control and Prediabetes groups. AntiLP-MedUse is substantially greater in T2DM patients (67.4%) compared to Prediabetes (34.6%) and Control (41.1%) groups. *Post-hoc* comparisons reveal substantial differences between the Control and T2DM groups (*p* < 0.001) and the Prediabetes group. QTd-10 s and QTd-5 min are ECG features with inconsistent results. QTd-10 s is statistically different among groups, whereas QTd-5 min is not. QRS-Axis-10 s differs considerably, especially between Control and T2DM, supporting ECG findings for diabetes categorization. In contrast, age and gender do not significantly affect diabetes classification in this population. OtherMeds-Depression is significantly different, suggesting an association between antidepressant use and diabetes status. *Post-hoc* comparisons show that Control vs. T2DM shows greater differences, while Prediabetes vs. T2DM does not for anti-depressive medication use.

Shorter ECG recordings improve categorization accuracy, according to the study. Adding the T2DM treatment feature enhances classification accuracy across all groups, boosting biomarker predictive power and model resilience (accuracy up to 0.88 ± 0.04). This suggests that medication data complements, rather than duplicates, diabetes status information. Medication use improved classification accuracy for patients without comorbidities, hypertensive patients, those with cardiovascular disease, and those with both conditions. These findings demonstrate that ECG biomarkers and medication data can be combined to improve the classification of diabetes.

### 3.3 Classification and feature selection by combining 10 s and 5 min ECG biomarkers

**Figure 5:**
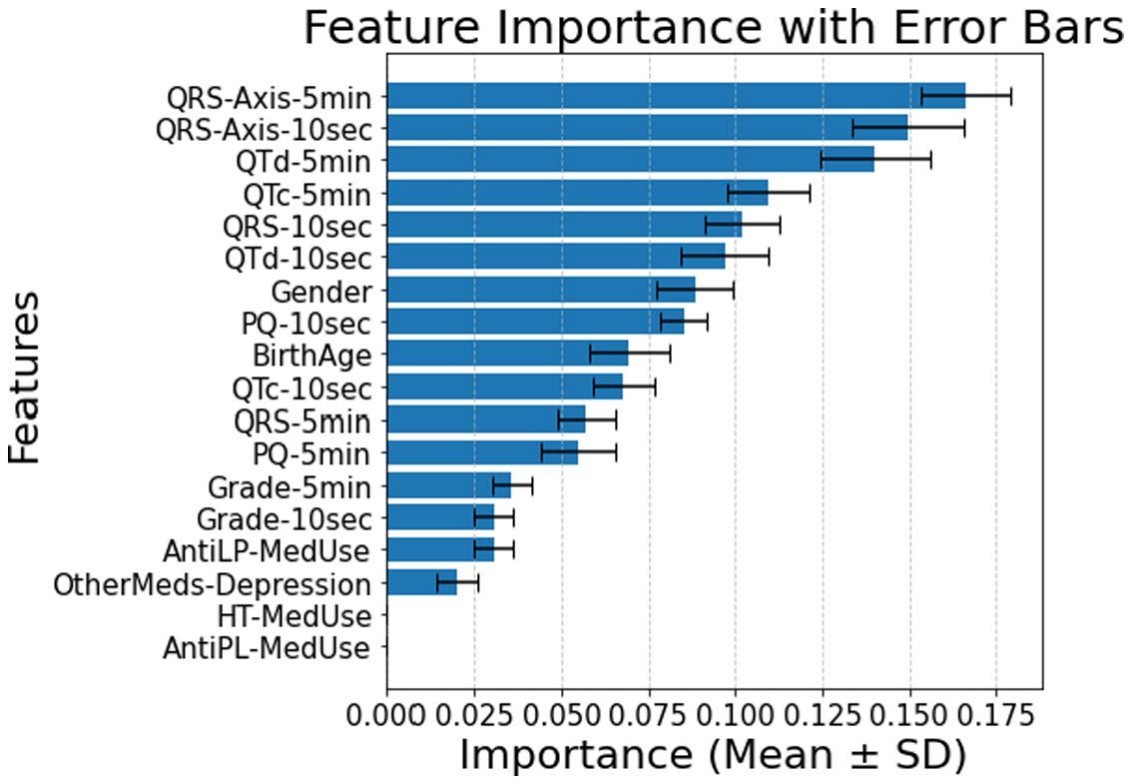
Feature importance for patients without comorbidity using all features for 10 s and 5 min. AntiLP-MedUse, Antilipid medication use; AntiPL-MedUse, Antiplatelet medication use; HT-MedUse, Hypertension medication use; PQ-10 s, PQ interval measured over 10 s; PQ-5 min, PQ interval measured over 5 min; QRS-10 s, QRS complex duration measured over 10 s; QRS-5 min, QRS complex duration measured over 5 min; QRS-Axis-10 s, QRS axis deviation measured over 10 s; QRS-Axis-5 min, QRS axis deviation measured over 5 min; QTc-10 s, Corrected QT interval measured over 10 s; QTc-5 min, Corrected QT interval measured over 5 min; QTd-10 s, QT dispersion measured over 10 s; QTd-5 min, QT dispersion measured over 5 min.

The classification of diabetes status in patients without comorbidities was primarily influenced by QRS-Axis-5 min, QRS-Axis-10 s, QTd-5 min, and QTc-5 min, with a classification accuracy of 0.91 ± 0.05 (Figure 5).

**Figure 6:**
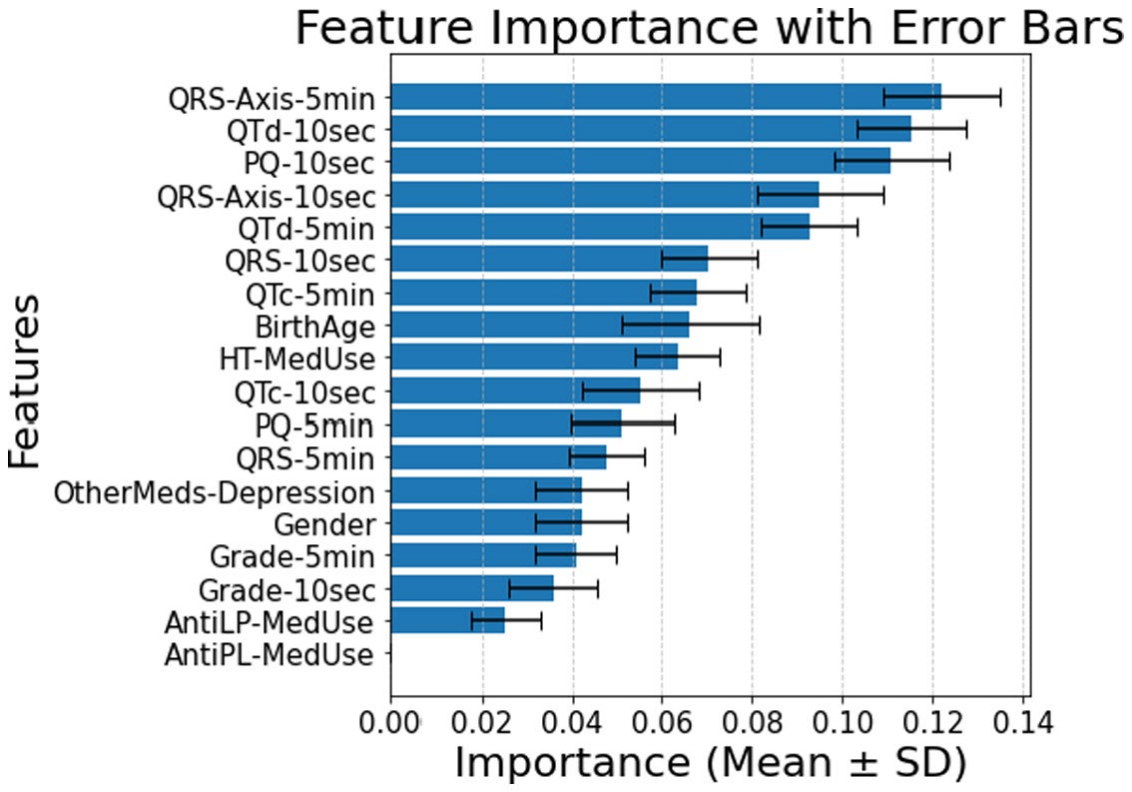
Feature importance for patients with hypertension using all features for 10 s and 5 min. AntiLP-MedUse, Antilipid medication use; AntiPL-MedUse, Antiplatelet medication use; HT-MedUse, Hypertension medication use; PQ-10 s, PQ interval measured over 10 s; PQ-5 min, PQ interval measured over 5 min; QRS-10 s, QRS complex duration measured over 10 s; QRS-5 min, QRS complex duration measured over 5 min; QRS-Axis-10 s, QRS axis deviation measured over 10 s; QRS-Axis-5 min, QRS axis deviation measured over 5 min; QTc-10 s, Corrected QT interval measured over 10 s; QTc-5 min, Corrected QT interval measured over 5 min; QTd-10 s, QT dispersion measured over 10 s; QTd-5 min, QT dispersion measured over 5 min.

For patients with HT, feature importance rankings shifted, with QRS-Axis-5 min, QTd-10 s, and PQ-10 s becoming the most influential features, and accuracy was 0.79 ± 0.07 (Figure 6).

**Figure 7:**
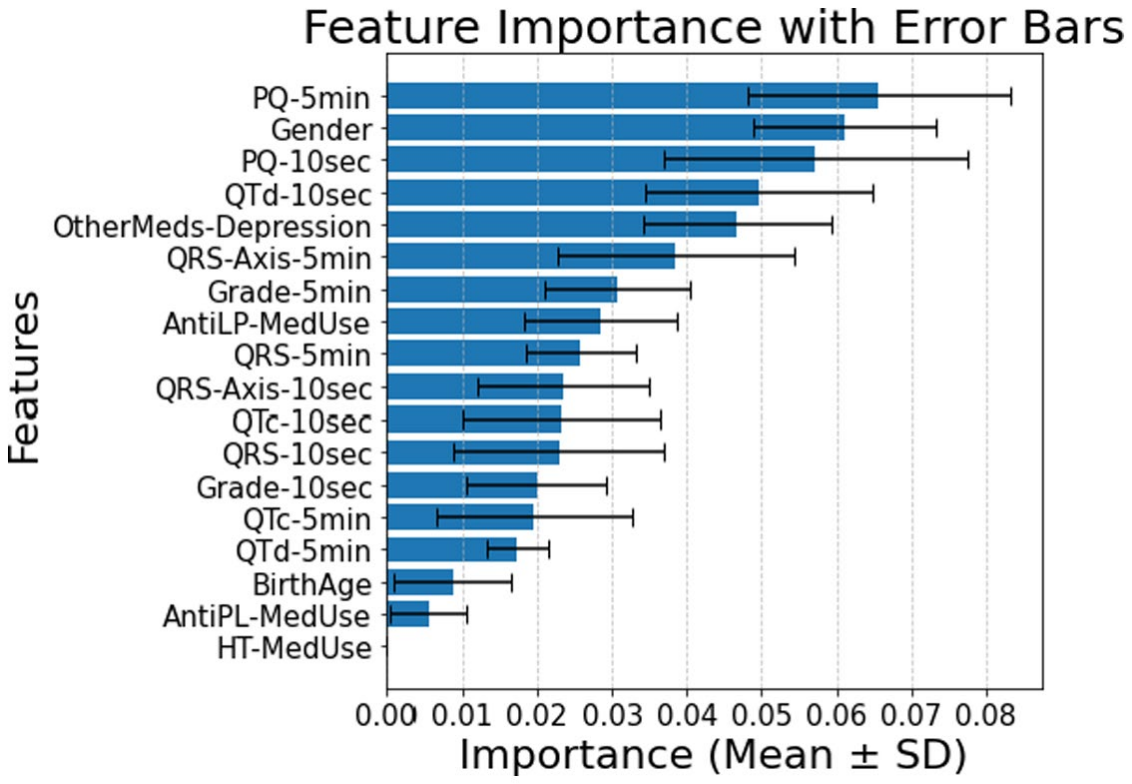
Feature importance for patients with cardiovascular disease using all features for 10 s and 5 min. AntiLP-MedUse, Antilipid medication use; AntiPL-MedUse, Antiplatelet medication use; HT-MedUse, Hypertension medication use; PQ-10 s, PQ interval measured over 10 s; PQ-5 min, PQ interval measured over 5 min; QRS-10 s, QRS complex duration measured over 10 s; QRS-5 min, QRS complex duration measured over 5 min; QRS-Axis-10 s, QRS axis deviation measured over 10 s; QRS-Axis-5 min, QRS axis deviation measured over 5 min; QTc-10 s, Corrected QT interval measured over 10 s; QTc-5 min, Corrected QT interval measured over 5 min; QTd-10 s, QT dispersion measured over 10 s; QTd-5 min, QT dispersion measured over 5 min.

In patients with CVD, PQ-5 min, Gender, and PQ-10 s emerged as the top predictors (Figure 7), leading to a classification accuracy of 0.82 ± 0.09.

**Figure 8:**
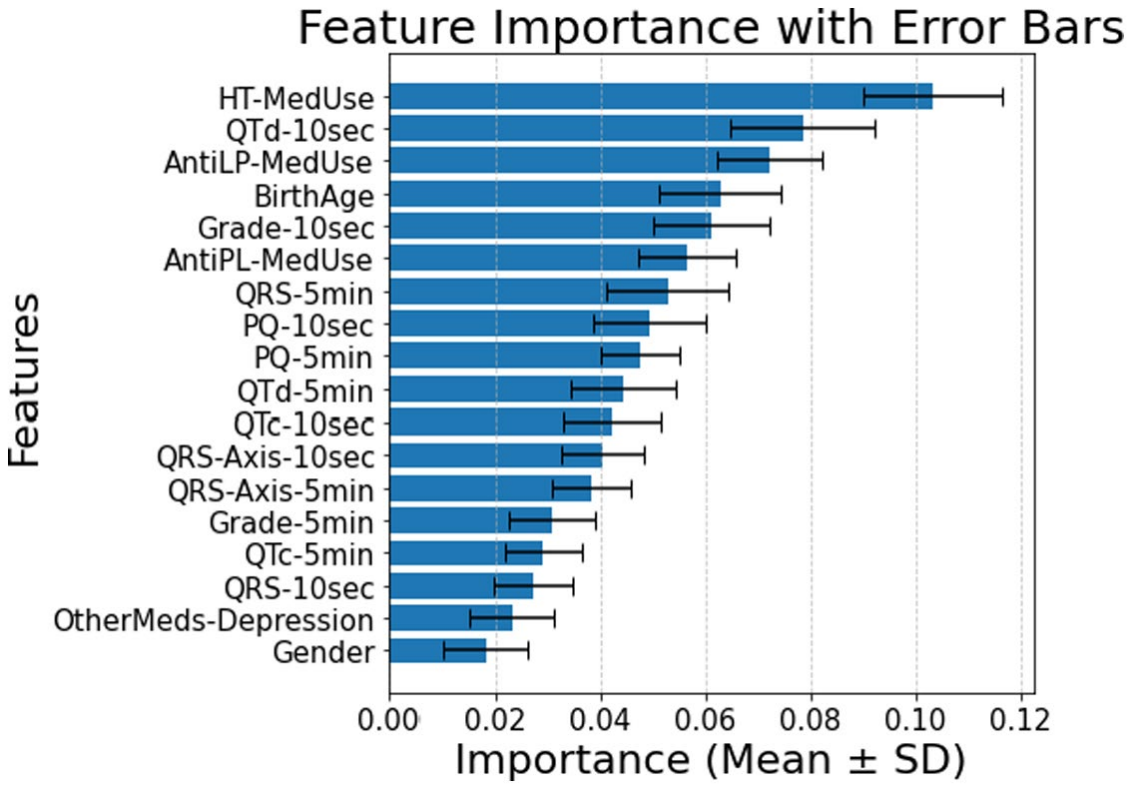
Feature importance for patients with both hypertension and cardiovascular disease using all features for 10 s and 5 min. AntiLP-MedUse, Antilipid medication use; AntiPL-MedUse, Antiplatelet medication use; HT-MedUse, Hypertension medication use; PQ-10 s, PQ interval measured over 10 s; PQ-5 min, PQ interval measured over 5 min; QRS-10 s, QRS complex duration measured over 10 s; QRS-5 min, QRS complex duration measured over 5 min; QRS-Axis-10 s, QRS axis deviation measured over 10 s; QRS-Axis-5 min, QRS axis deviation measured over 5 min; QTc-10 s, Corrected QT interval measured over 10 s; QTc-5 min, Corrected QT interval measured over 5 min; QTd-10 s, QT dispersion measured over 10 s; QTd-5 min, QT dispersion measured over 5 min.

For patients with both HT and CVD, antihypertensive medication use, QTd-10 s, and AntiLP-MedUse were the main features of the model, with a classification accuracy of 0.69 ± 0.07 (Figure 8). More detailed results of the performance metrics, including AUC per class, specificity, and sensitivity, are provided in the Supplementary material.

### 3.4 ECG biomarker dynamics in comorbidity groups without or with multiple medications

**Figure 9:**
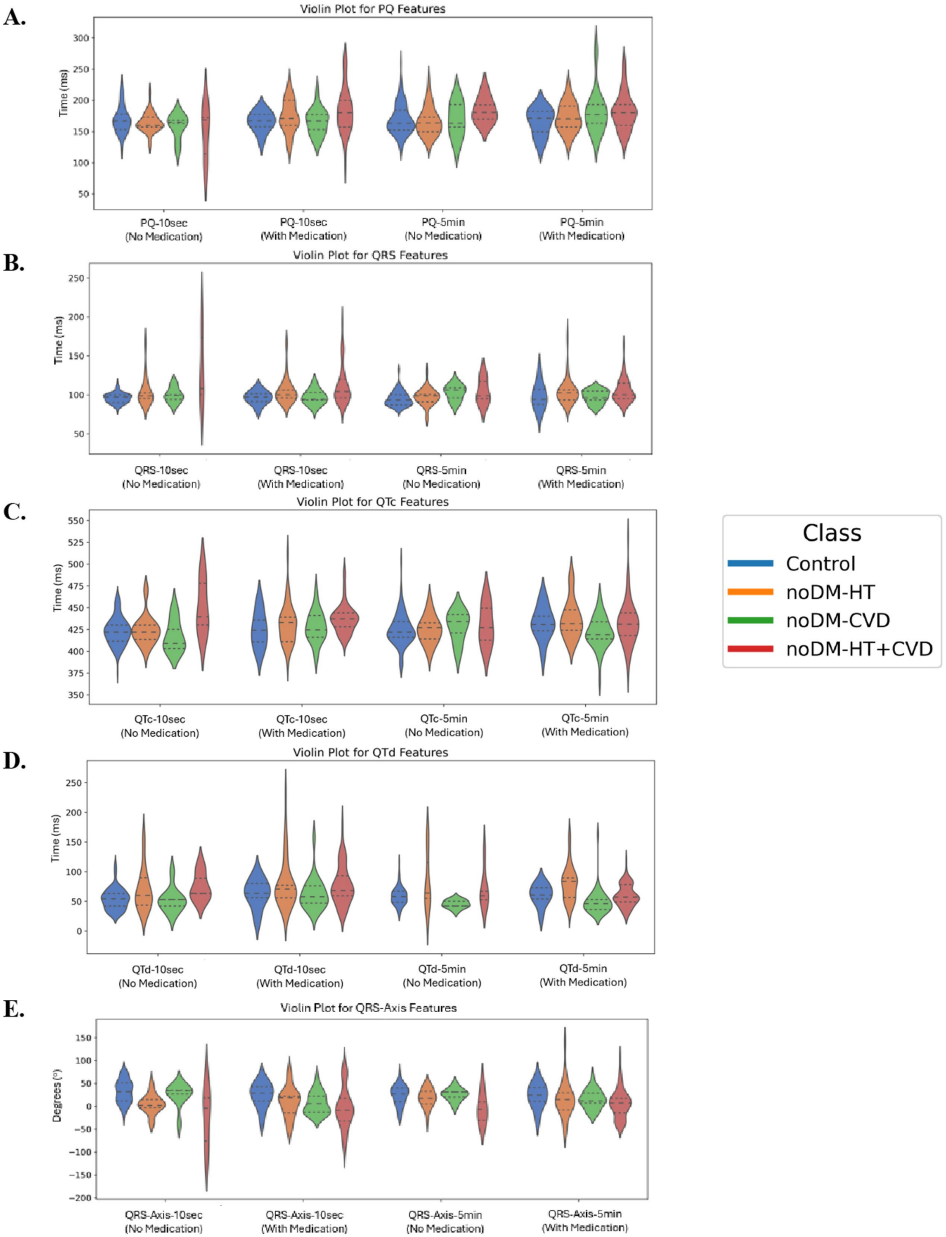
**(A)** PQ features for 10 s and 5 min, **(B)** QRS features for 10 s and 5 min, **(C)** QTc features for 10 s and 5 min, **(D)** QTd features for 10 s and 5 min, and **(E)** QRS-Axis features for 10 s and 5 min, for all control classes. All features show a statistically significant difference within the same time of measurement (Kruskal–Wallis test: *p* < 0.05). PQ-10 s, PQ interval measured over 10 s; PQ-5 min, PQ interval measured over 5 min; QRS-10 s, QRS complex duration measured over 10 s; QRS-5 min, QRS complex duration measured over 5 min; QRS-Axis-10 s. QRS axis deviation measured over 10 s; QRS-Axis-5 min, QRS axis deviation measured over 5 min; QTc-10 s, Corrected QT interval measured over 10 s; QTc-5 min, Corrected QT interval measured over 5 min; QTd-10 s, QT dispersion measured over 10 s; QTd-5 min, QT dispersion measured over 5 min.

The distributions of PQ, QRS, QTc, QTd, and QRS-Axis—measured over 10-s and 5-min intervals for all classes (healthy, noDM-HT, noDM-CVD, and noDM-HT + CVD), with and without medication, are presented in Figure 9. Across all features, significant differences were observed between the classes associated with either the 10-s ECG or 5-min ECG recordings, as determined by the Kruskal–Wallis test (*p* < 0.05). Notably, noDM-HT + CVD patients exhibited prolonged PQ intervals compared to the other groups, reflecting potential cardiac conduction delays in this group (Figure 9A). Healthy patients generally displayed shorter QRS intervals, whereas noDM-HT and noDM-HT + CVD patients showed wider QRS complexes, indicating ventricular conduction abnormalities in these groups (Figure 9B). The QTc features, with QTc-10 s and QTc-5 min, are prolonged in noDM-HT + CVD patients (Figure 9C). This suggests an elevated risk of arrhythmias in patients with combined hypertension and cardiovascular disease, which is consistent with clinical expectations given the known association between QTc prolongation and cardiovascular comorbidities. The dispersion of QT intervals (QTd) was higher in noDM-HT and noDM-HT + CVD groups, particularly the 10-s recordings (Figure 9D). This increased variability could indicate greater heterogeneity in ventricular repolarization in these populations. Finally, significant differences in QRS-Axis-10 s and QRS-Axis-5 min were observed among the control classes (Figure 9E). The noDM-HT and noDM-HT + CVD groups exhibited more negative QRS axes, indicative of left axis deviation, often associated with structural or electrical cardiac abnormalities.

**Figure 10:**
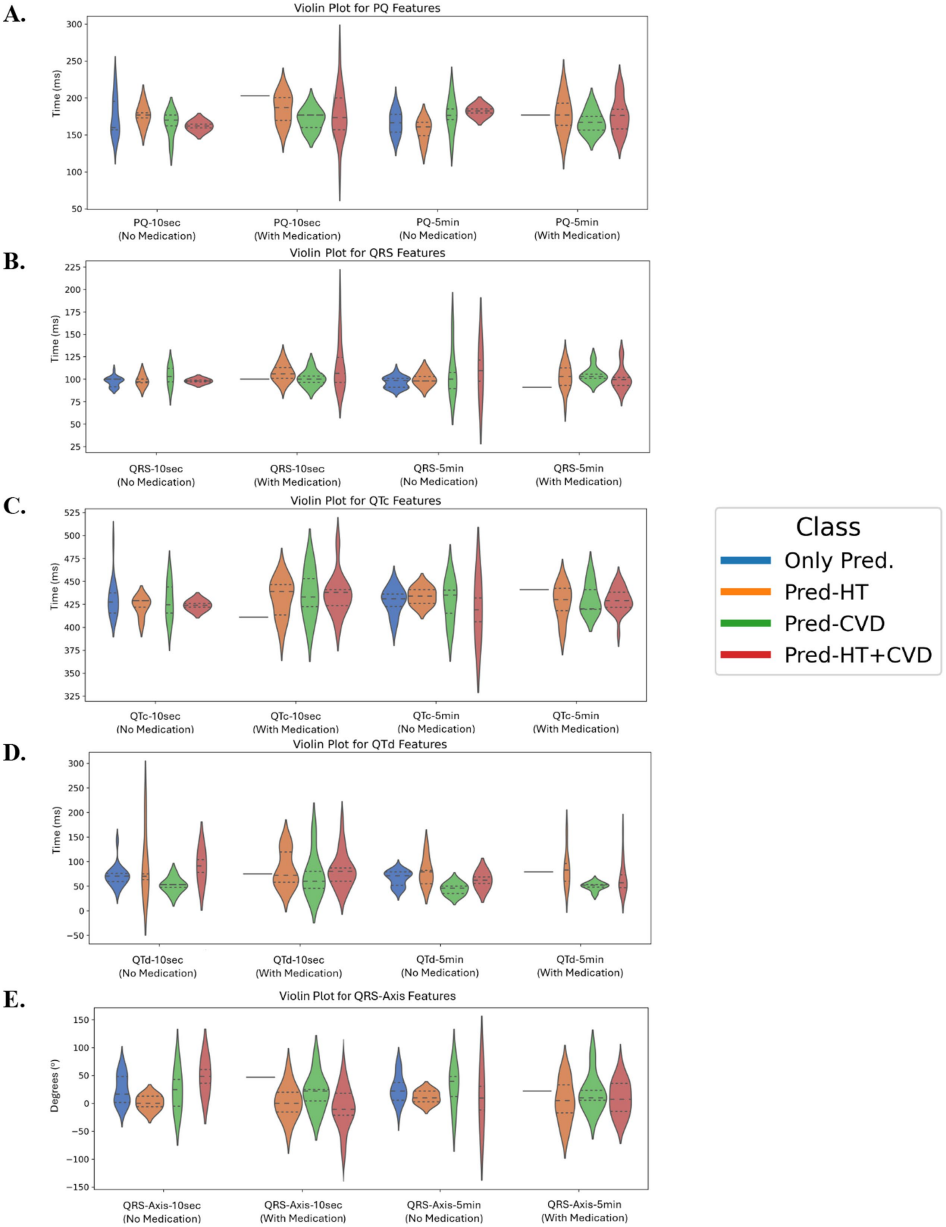
**(A)** PQ features for 10 s and 5 min, **(B)** QRS features for 10 s and 5 min, **(C)** QTc features for 10 s and 5 min, **(D)** QTd features for 10 s and 5 min, and **(E)** QRS-Axis features for 10 s and 5 min, for all prediabetes classes. All features show a statistically significant difference within the same time of measurement (Kruskal–Wallis test: *p* < 0.05). PQ-10 s, PQ interval measured over 10 s; PQ-5 min, PQ interval measured over 5 min; QRS-10 s, QRS complex duration measured over 10 s; QRS-5 min, QRS complex duration measured over 5 min; QRS-Axis-10 s, QRS axis deviation measured over 10 s; QRS-Axis-5 min, QRS axis deviation measured over 5 min; QTc-10 s, Corrected QT interval measured over 10 s; QTc-5 min, Corrected QT interval measured over 5 min; QTd-10 s, QT dispersion measured over 10 s; QTd-5 min, QT dispersion measured over 5 min.

The distributions of PQ, QRS, QTc, QTd, and QRS-Axis, measured over 10-s and 5-min intervals in all prediabetic subclasses (only prediabetes, prediabetes with HT, prediabetes with CVD, and prediabetes with HT + CVD) with and without medication, are presented in Figure 10. Class differences were significant within the same ECG measurement time, as shown by the Kruskal–Wallis test (*p* < 0.05). The PQ feature distributions show that PQ-10 s and PQ-5 min values differ between classes (Figure 10A). Prediabetes HT and HT + CVD, with medication, had longer PQ intervals than other classes, suggesting atrioventricular conduction delays. QRS features reveal distinct distributional differences across patient groups for both QRS-10 s and QRS-5 min durations (Figure 10B). Notably, the prediabetes groups with hypertension (HT) and with both HT and cardiovascular disease (CVD) exhibit broader and right-shifted distributions, indicating higher median and more variable QRS durations compared to the prediabetes-only group. These findings suggest potential abnormalities in cardiac conduction associated with comorbid HT and CVD. The prediabetes with CVD group is notably broader and skewed toward longer durations, particularly in QRS-5 min recordings. This indicates greater variability and a tendency toward prolonged QRS complexes, supporting the presence of conduction system impairments often associated with cardiovascular disease. Prediabetes CVD, and HT + CVD had longer QTc-10 s and QTc-5 min (Figure 10C), indicating a higher risk of ventricular arrhythmias in this sample. Only the prediabetes group had consistently shorter QTc intervals. QTd features show that prediabetes with HT and prediabetes with HT + CVD had larger QTd dispersion, especially over 10-s intervals, as in Figures 6, 10D. The ventricular repolarization in this group is more heterogeneous, explaining this diversity. QRS-Axis features show class differences over 10-s and 5-min intervals. Prediabetes with HT and prediabetes with HT + CVD groups had greater negative QRS-Axis values, indicating left axis deviation due to structural or electrical cardiac problems (Figure 10E).

**Figure 11:**
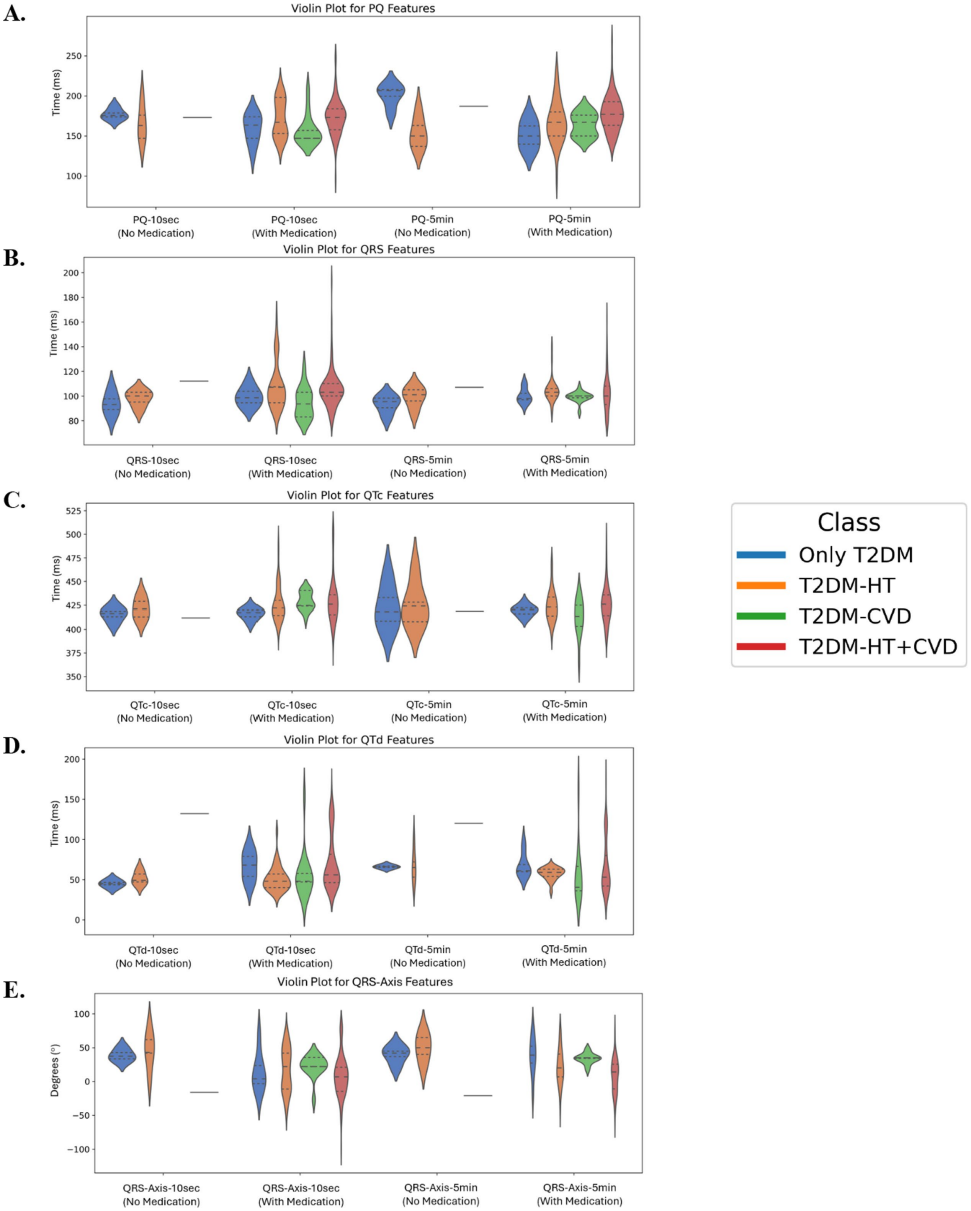
**(A)** PQ features for 10 s and 5 min, **(B)** QRS features for 10 s and 5 min, **(C)** QTc features for 10 s and 5 min, **(D)** QTd features for 10 s and 5 min, and **(E)** QRS-Axis features for 10 s and 5 min, for all T2DM classes. All features show a statistically significant difference associated with either the 10-s or 50 min recordings (Kruskal–Wallis test: *p* < 0.05). PQ-10 s, PQ interval measured over 10 s; PQ-5 min, PQ interval measured over 5 min; QRS-10 s, QRS complex duration measured over 10 s; QRS-5 min, QRS complex duration measured over 5 min; QRS-Axis-10 s, QRS axis deviation measured over 10 s; QRS-Axis-5 min, QRS axis deviation measured over 5 min; QTc-10 s, Corrected QT interval measured over 10 s; QTc-5 min, Corrected QT interval measured over 5 min; QTd-10 s, QT dispersion measured over 10 s; QTd-5 min, QT dispersion measured over 5 min.

PQ, QRS, QTc, QTd, and QRS-Axis feature distributions over 10-s and 5-min intervals in all T2DM classes (T2DM, T2DM with HT, T2DM with CVD, and T2DM with HT + CVD) with and without medication are shown in Figure 11. PQ interval results indicate that PQ-10 s and PQ-5 min periods differ across classes (Figure 11A). T2DM patients with HT and those with HT + CVD had longer PQ intervals than T2DM patients, suggesting atrioventricular conduction delays. Group differences in QRS-10 s and QRS-5 min durations are also present, as illustrated in Figure 11B. T2DM patients had shorter QRS intervals, but T2DM with HT and HT + CVD patients had wider QRS complexes, indicating ventricular conduction problems. QTc-10 s and QTc-5 min intervals are significantly prolonged in T2DM with HT and in patients with HT and CVD (Figure 11C), indicating a higher risk of arrhythmogenic events in this group. In contrast, T2DM patients had shorter QTc intervals. T2DM with CVD and HT + CVD had larger QTd dispersion (Figure 11D). The variability in ventricular repolarization was greatest when the 10-s recoding results were included. QRS-Axis characteristics in Figure 11E indicated substantial group differences in QRS-10 s and QRS-5 min. HT and HT + CVD patients had greater negative QRS-Axis values, indicating structural or electrical cardiac problems associated with left axis deviation.

## 4 Discussion

This study highlights the significant role of ECG biomarkers and medication use in classifying diabetes status, particularly in patients with HT and CVD. The widespread availability and use of ECGdiagnostics in primary care makes our findings clinically relevant. QTc, QTd, and QRS-Axis are reliable predictors of diabetes status, suggesting that ECG may be an effective, affordable, and non-invasive screening approach for early cardiac risk assessment in T2DM patients. Short-duration ECGs can be easily incorporated into routine patient assessment, or diabetes management to identify cardiovascular risk before symptoms appear. This is particularly important in primary care and low-income areas where comprehensive metabolic panels are not readily available for diagostics. Clinical approaches utilizing ECG-based risk assessment may facilitate rapid referrals, personalized monitoring, and early intervention (60, 61). By applying a machine learning model, we identified QRS-Axis, QTc, and QTd intervals as important ECG biomarkers, while lipid-lowering, and antihypertensive medications emerged as key distinguishing factors. Across all subgroups, QTc, QTd, and QRS-Axis consistently ranked among the top predictors of diabetes status. In patients without comorbidities, classification using these biomarkers achieved up to 91% accuracy. Even in treatment-naïve subgroups, ECG biomarkers retained predictive strength, supporting their utility for early screening. These findings align with existing research demonstrating that diabetes and cardiovascular conditions contribute to alterations in cardiac conduction and repolarization (62, 63).

The significant differences in QTd-10 s and QTc-5 min emphasize the importance of QRS-axis and QT-related characteristics in diabetes classification and that 10-s or 5-min recordings often provide different classification outcomes. Prior studies have linked QTc prolongation and increased QTd to heightened arrhythmic risk in diabetic populations (64). Our work extends these findings by demonstrating that machine learning-based classification models benefit from prioritizing ECG parameters in combination with medication use for optimal diabetes risk stratification. Beyond the methodological advancement of machine learning, our study provides clinical insights by identifying interactions between medication use and ECG biomarkers (65–67).

Our findings suggest that the interactions of medications, particularly anti-HT, DM-medication, and antidepressants, contribute to variations in ECG biomarkers, influencing diabetes classification performance. The prolongation of PQ intervals and widening of QRS complexes observed in diabetic patients with HT + CVD supports previous studies showing that antihypertensive drugs, particularly beta-blockers and calcium channel blockers, can prolong PR intervals and alter QRS duration (64). Moreover, we observed that QTc prolongation remained significant even in medicated patients, suggesting that standard cardiovascular treatments do not fully mitigate repolarization abnormalities in T2DM.

Statins are frequently prescribed and generally associated with cardioprotective effects but did not show significant associations with ECG alterations in our study, suggesting that their cardiovascular benefits may not directly impact ECG patterns (63). Similarly, antidepressant medications showed limited influence on diabetes classification, consistent with prior research indicating that SSRIs have a lower impact on ECG compared to tricyclic antidepressants (TCAs) (68). However, the statistically significant association between antidepressant use and diabetes in our study suggests that psychiatric conditions may play an indirect role in metabolic and cardiovascular risk, warranting further investigation.

Notably, the lowest accuracy (0.64) was observed in the group with hypertension and cardiovascular disease, which is acceptable given the expected patient heterogeneity in this subgroup. Previous studies have reported variability in ECG alterations based on antidiabetic medication, such as GLP-1 receptor agonists and SGLT2 inhibitors (62). Our results indicate that excluding DM medication data allows ECG biomarkers to play a more prominent role in classification. Our study aligns with prior research demonstrating that QTc and QTd prolongation are hallmarks of diabetes-related cardiac risk. However, few studies have systematically examined the impact of medication use on ECG biomarker variability in diabetes classification models. Unlike prior studies focusing solely on ECG alterations, our work integrates machine-learning approaches to quantify the importance of ECG biomarkers and medication use. This provides a novel perspective on how medication effects interact with cardiac conduction in diabetic patients. Existing studies have also suggested that patients with multiple comorbidities (HT + CVD) exhibit the most pronounced ECG abnormalities (64). Our findings corroborate this, showing that PQ-10 s, QRS-Axis-10 s, and QTc-10 s were significantly altered in these patients.

Our analysis shows that ECG recording duration significantly impacts machine learning-based diabetes classification accuracy. Some 5-min ECG recordings showed higher accuracies due to their ability to capture more cardiac variability, autonomic function, and repolarization instability. These findings support previous research that longer ECG interval recordings may be needed for risk assessment when a diabetes history is uncertain. Although the accuracy was higher when using 5-min ECG features, 10-s recordings remained predictive, supporting their potential use in diabetes screening. Short ECGs, which are easily obtainable, may be useful for early cardiac risk stratification in diabetic patients, particularly in outpatient and primary care settings. The high classification accuracy of 5-min ECGs without suggesting that prolonged monitoring captures subtle electrophysiological variations linked to metabolic status and cardiovascular risk, making up for the missing treatment-related information. This supports previous findings that longer ECG durations improve assessments of repolarization abnormalities, which are common in diabetes and are associated with arrhythmias. These findings are clinically significant. When diabetes history is unavailable or incomplete, 5-min ECGs with long-term cardiac dynamics improve classification accuracy.

Although SMOTE introduces synthetic samples, its application was necessary to mitigate the substantial class imbalance across the patient subgroups, particularly for patients without comorbidities or medication use. To ensure that this oversampling did not introduce artificial or non-physiological patterns, we evaluated model robustness using 5-fold cross-validation for the 10-s and 5-min ECG recordings and classifiers. Classification accuracy (~0.70–0.91) remained consistent, with QTc, QTd, and PQ intervals contributing to all models. These features are well-established in cardiovascular physiology, particularly in diabetes-related arrhythmic risk, which supports the physiological validity of our results (33, 52, 69). Medication-induced ECG changes (e.g., QTc prolongation with antidepressants or PQ changes with antihypertensives) corresponded with established electrophysiological causes, confirming that the patterns observed were not synthetic oversampling.

The medication usage improved classification accuracy but may also add bias, confusing ECG biomarker contributions independent of treatment effects. To explore the possibility of medication-induced bias in ECG interpretation, the treatment-naïve subgroups, i.e., patients without any medication, were analyzed. In these subgroups, QTd-10 s, PQ-10 s, and QRS-Axis-10 s were consistently ranked among the top features, with classification accuracies of 0.86 and 0.88 for 10-s and 5-min recordings, respectively (70, 71). When medication variables were more present in groups with comorbidities (HT, CVD, and HT + CVD), the permutation importance revealed that both key ECG and medication features remained more predictive or equally important. These findings indicate that medication status does not affect biomarker interpretation but enhances precision by distinguishing disease-related from pharmacologically related ECG changes.

Exclusion criteria were relaxed to match real-world screening conditions where individuals had several comorbidities and continued medication use. This design makes ECG biomarker evaluation in varied clinical groups more practical. But it introduces possible confounders and increases unpredictability. While our dataset originates from an Australian clinical screening population, several factors support its broader applicability. The ECG dataset was previously validated through peer-reviewed studies using spatial modeling and clustering to assess the representativeness of larger rural and regional populations (72–74). Our model uses clinically universal ECG biomarkers that are commonly used in clinical cardiology. Additionally, our investigation examined ECG biomarkers and medication use, rather than HbA1c, body mass index (BMI), lipid profiles, or diabetes duration. These metrics can adjust for confounders, give metabolic and physiological context, and improve ECG biomarker interpretation. Clinical and biochemical markers should be included in future studies as well as test the model on diverse populations to improve diagnostics and risk classification. Future studies should examine whether ECG-based risk assessment, combined with continuous glucose monitoring, heart rate variability, or blood pressure variability, can be applied in larger multiethnic groups to enhance diagnostic performance. Our findings so far demonstrate that short ECGs can be easily integrated into routine healthcare workflows to detect diabetes-related cardiac risk early.

This study highlights the clinical relevance of ECG-based screening in diabetes management, particularly for patients with hypertension and cardiovascular disease, to detect early conduction abnormalities. While antihypertensive and lipid-lowering drugs may help stabilize cardiac function, their effects on QTc and QTd warrant further investigation. Machine learning models that incorporate ECG biomarkers and medication history can enhance diabetes classification and facilitate early risk detection. Future research should investigate the effects of specific antidiabetic agents on ECG parameters and evaluate alternative treatments to mitigate cardiac complications in T2DM. Additionally, longitudinal studies are needed to examine how ECG biomarkers evolve over time in diabetic patients and how treatment regimens influence these changes. Investigating gender-based differences in diabetes-related ECG alterations could also provide new insights, as our findings suggest higher QRS-Axis variability in male patients with prediabetes (*p* = 0.006).

## 5 Conclusion

In this study, comorbidities, medication use, and cardiac electrophysiological changes are examined with type 2 diabetes, prediabetes, and healthy controls. Integrating ECG biomarkers with pharmacological profiles, including antihypertensive, lipid-lowering, antiplatelet, and depression-specific medications, provided our machine learning models with good classification accuracy. This highlights the importance of physiological and treatment-related diabetes risk stratification. T2DM patients with hypertension and cardiovascular disease showed the most ECG abnormalities, including longer QTc, QTd, and QRS-Axis, suggesting arrhythmogenic and conduction risks. Five-minute ECG records were more predictive, but 10-s recordings were good for diabetes classification, indicating their application in scalable screening systems. These findings suggest that rapid, non-invasive ECG tests and advanced analytics improve early diabetes detection, disease progression, and personalized treatment. Future research should evaluate the advantages of combining ECG data with continuous glucose monitoring, heart rate variability, or wearable device outputs in larger multiethnic groups. The electrophysiological impact of diabetes therapy may be revealed by studying how GLP-1 receptor agonists and SGLT2 inhibitors influence ECG markers.

## Data Availability

The raw data supporting the conclusions of this article will be made available by the authors. Please contact the corresponding author for available data.
