# Supplementary material for: AI-based integration of ECG biomarkers for assessing cardiac risk in type 2 diabetes mellitus with comorbid conditions for patient stratification
Source: Front Med (Lausanne). 2025 Sep 8;12:1646495. doi: 10.3389/fmed.2025.1646495 (PMC12450978; doi:10.3389/fmed.2025.1646495)
Supplement: Supplementary file 1 [file Data_Sheet_1.docx]

**Supporting Information**

**Contents**

[1. Introduction 2](#_Toc203373213)

[2. Performance metrics for patients without any comorbidity 3](#_Toc203373214)

[3. Performance metrics for patients with HT 6](#_Toc203373215)

[4. Performance metrics for patients with CVD 9](#_Toc203373216)

[5. Performance metrics for patients with HT and CVD 12](#_Toc203373217)

# 1. Introduction

Differentiation between patients with and without comorbidities in Type 2 diabetes mellitus (T2DM) is quite important. ECG biomarkers identify phenotypes non-invasively; however, their diagnostic value depends on whether hypertension (HT), cardiovascular disease (CVD), or both are present. This report includes more classification metrics for our models for individuals without comorbidities, with hypertension (HT) only, with cardiovascular disease (CVD) solely, and concurrent HT and CVD. We compared 10 seconds, 5 minutes, and a combination of both ECG recordings for effectiveness. Model performance was measured by AUC per class, specificity, and sensitivity.

# 2. Performance metrics for patients without any comorbidity


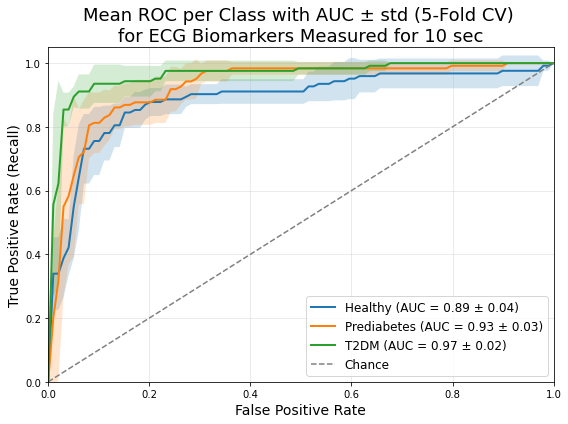


**Figure S.1:** Mean ROC per class for patients without comorbidities for ECG biomarkers measured for 10 seconds.


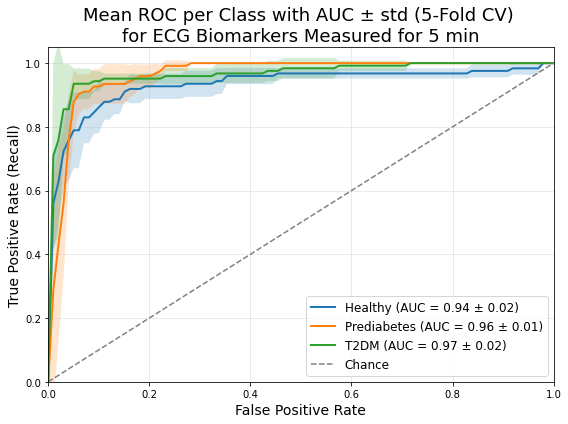


**Figure S.2:** Mean ROC per class for patients without comorbidities for ECG biomarkers measured for 5 minutes.


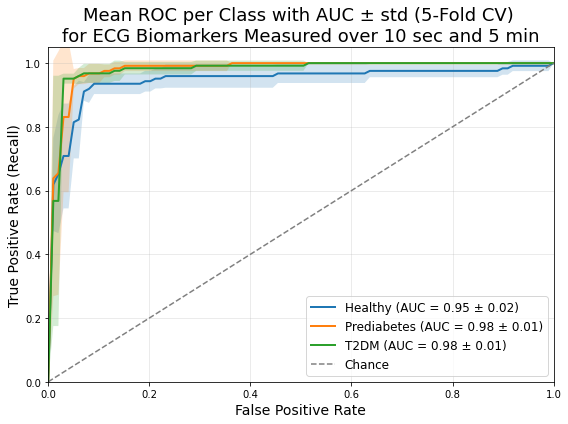


**Figure S.3:** Mean ROC per class for patients without comorbidities for ECG biomarkers measured over 10 seconds and 5 minutes.

**Table S.1:** Specificity results for patients without any comorbidity

| **Class** | **ECG with**  **10 sec features** | **ECG with**  **5 min features** | **ECG with both 10 sec and**  **5 min features** |
| --- | --- | --- | --- |
| **Healthy** | 0.91±0.03 | 0.94±0.02 | 0.96±0.02 |
| **Prediabetes** | 0.91±0.03 | 0.91±0.03 | 0.95±0.02 |
| **T2DM** | 0.93±0.02 | 0.91±0.03 | 0.96±0.02 |

**Table S.2:** Sensitivity results for patients without any comorbidity

| **Class** | **ECG with**  **10 sec features** | **ECG with**  **5 min features** | **ECG with both 10 sec and**  **5 min features** |
| --- | --- | --- | --- |
| **Healthy** | 0.74±0.06 | 0.76±0.09 | 0.84±0.06 |
| **Prediabetes** | 0.84±0.07 | 0.91±0.05 | 0.95±0.03 |
| **T2DM** | 0.93±0.06 | 0.93±0.03 | 0.95±0.02 |

In 10 seconds, 5 minutes, or both, ECG markers can identify healthy, prediabetic, and T2DM patients without comorbidities. All three classes achieved high classification accuracy, with AUC values improving when 10-second and 5-minute data were integrated. High-class separability is demonstrated by the combined model's AUCs of 0.95 for healthy individuals, 0.98 for prediabetes, and 0.98 for T2DM (Figs. S.1-S.3).

Specificity improved with more ECG data, from 0.91 (10 sec) to 0.96 (combined) for the healthy group. Also, specificity increased from 0.91 to 0.95 in prediabetes and 0.93 to 0.96 in T2DM (Table S.1). Short and lengthy ECG characteristics may help models avoid incorrect diagnoses, especially for borderline or early-stage patients.

The T2DM group exhibited the highest true positive rate (sensitivity) across all ECG durations (Table S.2), 0.95 in the combined model. The model appears to accurately detect diabetes. The sensitivity of prediabetes increased from 0.84 (10 sec) to 0.95 (combined), demonstrating the temporal scale's ability to detect modest cardiac changes in early diabetic dysregulation. The healthy group exhibited the lowest sensitivity (0.74 at 10 sec), but the combined features increased it to 0.84. Healthy patients have higher cardiac signature variability, and longer ECG data increases classification robustness.

All diabetic classes have been enhanced in specificity and sensitivity using 10-second and 5-minute ECG indications in healthy individuals. Short ECGs could diagnose T2DM, but longer ones improved diagnostic balance, notably for prediabetes and healthy controls. These results corroborate ECG-based models for early hyperglycemia classification and suggest that combined-duration ECG analysis may be more applicable in real-world screening settings.

# 3. Performance metrics for patients with HT


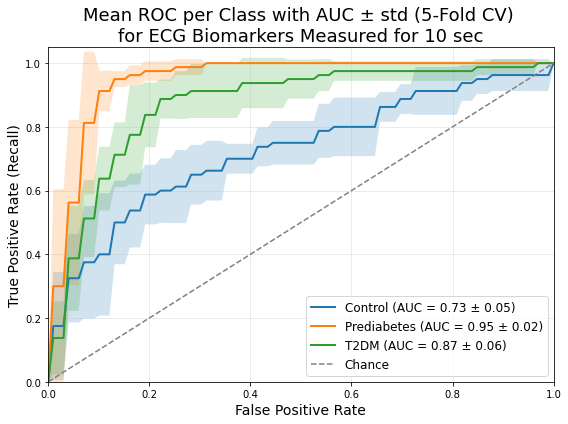


**Figure S.4:** Mean ROC per class for patients with HT for ECG biomarkers measured for 10 seconds.


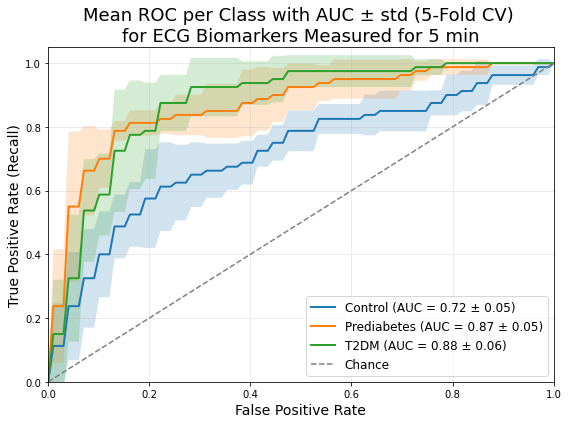


**Figure S.5:** Mean ROC per class for patients with HT for ECG biomarkers measured for 5 minutes.


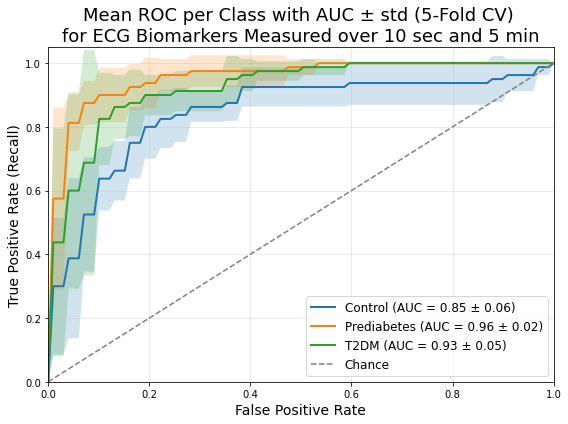


**Figure S.6:** Mean ROC per class for patients with HT for ECG biomarkers measured over 10 seconds and 5 minutes.

**Table S.3:** Specificity results for patients with HT

| **Class** | **ECG with**  **10 sec features** | **ECG with**  **5 min features** | **ECG with both 10 sec and**  **5 min features** |
| --- | --- | --- | --- |
| **Control** | 0.83±0.03 | 0.81±0.05 | 0.89±0.05 |
| **Prediabetes** | 0.91±0.02 | 0.88±0.04 | 0.89±0.07 |
| **T2DM** | 0.85±0.05 | 0.85±0.04 | 0.91±0.06 |

**Table S.4:** Sensitivity results for patients with HT

| **Class** | **ECG with**  **10 sec features** | **ECG with**  **5 min features** | **ECG with both 10 sec and**  **5 min features** |
| --- | --- | --- | --- |
| **Control** | 0.56±0.11 | 0.56±0.06 | 0.69±0.10 |
| **Prediabetes** | 0.88±0.06 | 0.75±0.04 | 0.89±0.07 |
| **T2DM** | 0.74±0.11 | 0.78±0.10 | 0.83±0.10 |

The classification performance of HT patients utilizing ECG biomarkers from short (10 sec) and extended (5 min) records is strong (Figs. S.4-S.6). Prediabetes and T2DM show good AUC values across all durations, but the control class improves significantly with feature combination. When 10-second and 5-minute ECG characteristics are combined, AUC values reach 0.85 for the control group, 0.96 for the prediabetes group, and 0.93 for the T2DM group, demonstrating that ECG-based classification is effective even in the presence of hypertensive comorbidity.

All classes exhibit high specificity (Table S.3), notably for prediabetes and T2DM, where the model accurately identifies non-cases. Specificity for prediabetes is 0.92 (10 sec), 0.88 (5 min), and 0.89. At 0.85–0.91, the specificity of T2DM remains robust over time. While control group specificity is significantly lower, feature combination improves it (from 0.83 to 0.89). The model appears to flag false positives more conservatively.

Unlike specificity, sensitivity (Table S.4) is lower, notably in the control group, where sensitivity is 0.69 or lower across all configurations. The model may misclassify some healthy patients as prediabetic or diabetic due to a greater probability of false negatives. Using more extensive ECG characteristics enhances T2DM sensitivity from 0.74 (10 sec) to 0.83 (combined).

Prediabetes sensitivity decreases in the 5-minute model (0.75) compared to the 10-second model (0.88), but improves in the combined model (0.89). Hypertension may obscure or overlap cardiac signs, especially in normoglycemic patients, reducing sensitivity. Hypertension alters cardiac electrophysiology, which may cause ECG changes similar to those found in prediabetic or diabetic patients, thereby limiting the model's capacity to distinguish between healthy instances.

ECG-based T2DM classification is feasible and useful despite the presence of hypertension-related cardiac alterations. Specificity is high across classes, and coupled ECG lengths boost sensitivity, especially for prediabetes and T2DM.

4. Performance metrics for patients with CVD


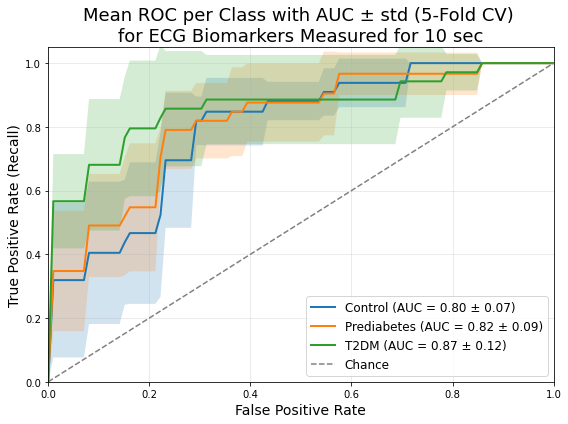


**Figure S.7:** Mean ROC per class for patients with CVD for ECG biomarkers measured for 10 seconds.


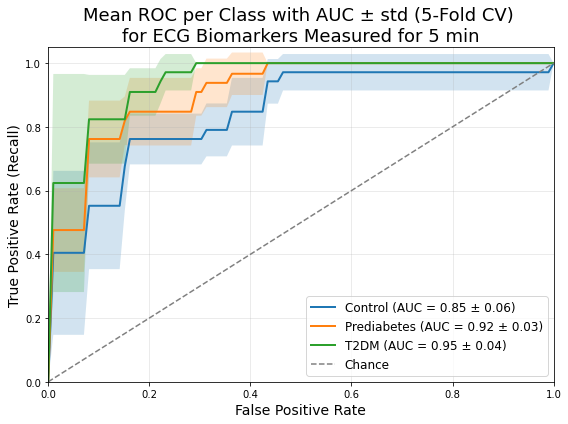


**Figure S.8:** Mean ROC per class for patients with CVD for ECG biomarkers measured for 5 minutes.


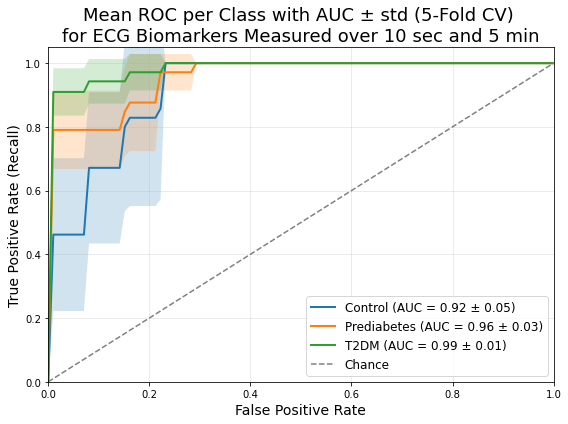


**Figure S.9:** Mean ROC per class for patients with CVD for ECG biomarkers measured over 10 seconds and 5 minutes.

**Table S.5:** Specificity results for patients with CVD

| **Class** | **ECG with**  **10 sec features** | **ECG with**  **5 min features** | **ECG with both 10 sec and**  **5 min features** |
| --- | --- | --- | --- |
| **Control** | 0.76±0.09 | 0.91±0.03 | 0.87±0.09 |
| **Prediabetes** | 0.80±0.12 | 0.75±0.09 | 0.90±0.06 |
| **T2DM** | 0.88±0.06 | 0.91±0.07 | 0.96±0.06 |

**Table S.6:** Sensitivity results for patients with CVD

| **Class** | **ECG with**  **10 sec features** | **ECG with**  **5 min features** | **ECG with both 10 sec and**  **5 min features** |
| --- | --- | --- | --- |
| **Control** | 0.59±0.17 | 0.55±0.15 | 0.73±0.11 |
| **Prediabetes** | 0.59±0.04 | 0.82±0.13 | 0.82±0.12 |
| **T2DM** | 0.71±0.20 | 0.76±0.19 | 0.89±0.11 |

Combining short (10-second) and prolonged (5-minute) ECG recordings improves CVD categorization using ECG biomarkers. ROC plots and AUC values show that the integrated feature model classifies well, with AUC values of 0.92 for the control group, 0.96 for prediabetes, and 0.99 for T2DM. In contrast, ECG characteristics from a single time scale reduce AUC values, especially for control and prediabetes (Figs. S.7-S.9). The AUC for prediabetes is 0.82 and T2DM is 0.87 with 10-second ECGs, but 0.92 and 0.95 with 5-minute ECGs. The findings show that ECG biomarker patterns over several time scales are superior for diagnosing CVD patients' diabetic state.

High specificity is shown across all classes and ECG configurations (Table S.5). Specificity for T2DM ranges from 0.88 (10 sec) to 0.96 (combined). This suggests the algorithm accurately identifies diabetics in the CVD sample. The specificity of prediabetes increases from 0.80 (10 seconds) to 0.90 (combined), whereas the specificity of the control group increases from 0.76 to 0.87. The higher temporal resolution and variability of integrated ECG characteristics may help distinguish overlapping abnormalities in this complicated clinical group.

Sensitivity varies considerably among courses and durations (Table S.6). The control group has poor sensitivity (0.59 ± 0.17 for 10-second features, 0.55 ± 0.15 for 5-minute features) but increases to 0.73 ± 0.11 when both are merged. This shows that the model struggles to identify healthy CVD patients due to overlapping ECG abnormalities caused by the other groups. In the prediabetes group, sensitivity increases from 0.59 (10 sec) to 0.82 (5 min) and remains robust at 0.82 in the combined model. T2DM patients range from 0.71 (10 sec) to 0.89 (combined). The control group's reduced sensitivity may be due to technical reasons, such as modest ECG alterations in prediabetes and early T2DM overlapping with CVD-induced changes in the control group, making it difficult for the model to distinguish diabetic classes from ECG inputs.

5. Performance metrics for patients with HT and CVD


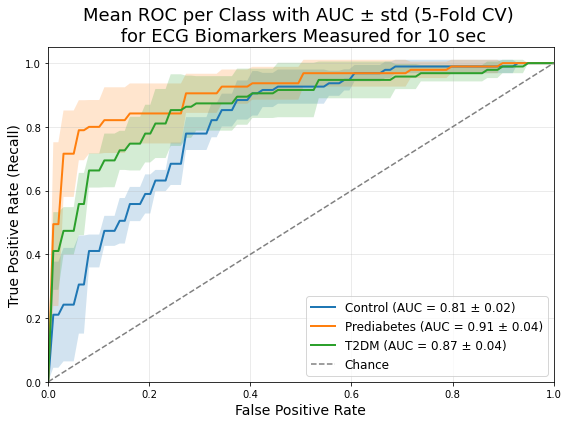


**Figure S.10:** Mean ROC per class for patients with both HT and CVD for ECG biomarkers measured for 10 seconds.


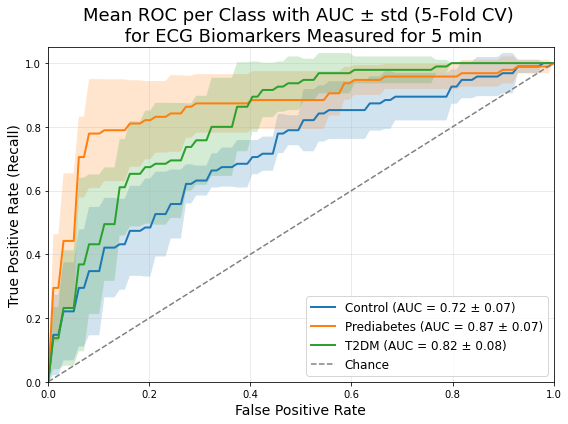


**Figure S.11:** Mean ROC per class for patients with both both HT and CVD for ECG biomarkers measured for 5 minutes.


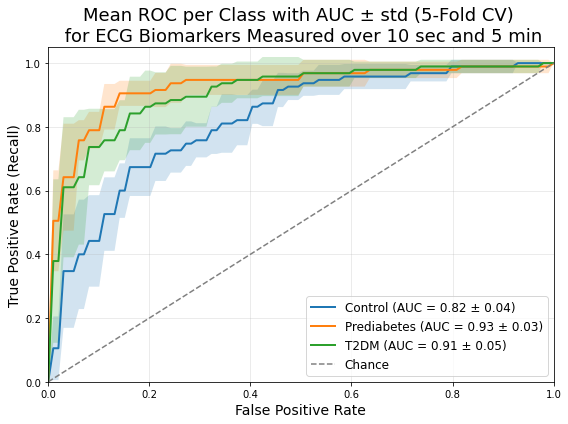


**Figure S.12.:** Mean ROC per class for patients with both hypertension and cardiovascular disease for ECG biomarkers measured over 10 seconds and 5 minutes.

**Table S.7:** Specificity results for patients with both hypertension and cardiovascular disease

| **Class** | **ECG with**  **10 sec features** | **ECG with**  **5 min features** | **ECG with both 10 sec and**  **5 min features** |
| --- | --- | --- | --- |
| **Control** | 0.82±0.06 | 0.83±0.10 | 0.86±0.03 |
| **Prediabetes** | 0.86±0.03 | 0.86±0.06 | 0.87±0.06 |
| **T2DM** | 0.85±0.08 | 0.79±0.09 | 0.94±0.05 |

**Table S.8:** Sensitivity results for patients with both hypertension and cardiovascular disease

| **Class** | **ECG with**  **10 sec features** | **ECG with**  **5 min features** | **ECG with both 10 sec and**  **5 min features** |
| --- | --- | --- | --- |
| **Control** | 0.55±0.10 | 0.52±0.10 | 0.71±0.11 |
| **Prediabetes** | 0.80±0.08 | 0.77±0.15 | 0.88±0.08 |
| **T2DM** | 0.72±0.08 | 0.68±0.17 | 0.76±0.11 |

In patients with both HT and CVD, ECG biomarkers can classify diabetic state, with 10-second and 5-minute characteristics improving accuracy (Figs. S.10-S.12). ROC graphs reveal that the integrated model has high AUC values: 0.82 for control, 0.93 for prediabetes, and 0.91 for T2DM (Fig. S.12). Even in a clinically complicated group, our data show strong diabetic class separability. However, utilizing ECG characteristics from a single duration provides lower AUCs, especially for prediabetes and T2DM with 10-second ECGs (0.91 and 0.87, respectively) and the control group with 5-minute ECGs (0.72).

Specificity is excellent across all three classes and improves with feature combination (Table S.7). Both periods had 0.94 T2DM specificity, up from 0.85 (10 seconds) and 0.79 (5 minutes). Control rises from 0.82 to 0.86 and prediabetes specificity from 0.86 to 0.87. The model can effectively identify patients of each class, which is important in people with overlapping cardiovascular risk factors. Dual-duration ECG characteristics may reduce HT and CVD-induced baseline heart rhythm uncertainty.

Some classes have low sensitivity (Table S.8). The control group had the lowest sensitivity, improving from 0.55 (10 seconds) and 0.52 (5 minutes) to 0.71 (combined). Combining characteristics increases prediabetes sensitivity from 0.80 (10 seconds) and 0.77 (5 minutes) to 0.88. T2DM sensitivity ranges from 0.72 (10 sec) to 0.68 (5 min) and decreases to 0.76 in the combined model. The combined model enhances performance; however, some configurations have reduced control and T2DM sensitivity, which requires more evaluation.
